# Identifying and understanding the health-related quality of life outcomes experienced by solid organ transplant recipients: a systematic review of qualitative research

**DOI:** 10.1186/s41687-026-01158-2

**Published:** 2026-07-24

**Authors:** Ben Rimmer, Sarah Dickson, Siân Russell, Rebeka Jenkins, Dawn Craig, Linda Sharp, Catherine Exley

**Affiliations:** 1 Population Health Sciences Institute, Newcastle University, Newcastle upon Tyne, UK; 2 Statistics and Clinical Studies, NHS Blood and Transplant, Bristol, UK

**Keywords:** Solid organ transplant recipients, Health-related quality of life, Qualitative research

## Abstract

**Purpose:**

This review aimed to identify and understand the health-related quality of life (HRQoL) outcomes experienced post-transplant by solid organ transplant recipients.

**Methods:**

We systematically searched MEDLINE, Embase, CINAHL, PsycINFO, and Scopus from inception to 17th May 2024. Search hits were independently screened, then study and sample characteristics were extracted from eligible papers, and transparency of reporting was appraised. A best fit framework synthesis was conducted to identify and map HRQoL outcomes to nine pre-defined HRQoL dimensions, and synthesise cross-cutting patterns in peoples’ HRQoL experiences across studies.

**Results:**

We identified 40 papers reporting 39 qualitative studies of HRQoL in transplant recipients (kidney *n* = 14 studies, liver *n* = 10, heart *n* = 8, lung *n* = 3, mixed organs *n* = 4). Sample diversity (e.g. ethnicity, rurality) was lacking, or poorly reported, within and across included studies. Transparency of reporting was adequate. We identified 93 HRQoL outcomes; these could all be mapped to the nine HRQoL dimensions. The most commonly reported dimensions were emotional (*n* = 40 papers), social (*n* = 37), and role functioning (*n* = 35). Across solid organs, there were many common outcomes, encompassing positive and negative emotional impact, complex social and role challenges, and wide-ranging symptom distress.

**Conclusions:**

Solid organ transplant recipients experience extensive positive and negative HRQoL outcomes across every aspect of life, indicating where support may be required following transplantation. These findings inform a comprehensive and consolidated framework of the HRQoL outcomes experienced by transplant recipients. This provides valuable insights for the future development of transplant-specific patient-reported outcome measures, to ensure they comprehensively capture the HRQoL outcomes that are relevant to transplant recipients.

**Supplementary Information:**

The online version contains supplementary material available at https://doi.org/10.1186/s41687-026-01158-2.

## Abstract (plain language summary)

The number of people living long-term with an organ transplant is increasing. Health-related quality of life, that is a person’s physical, emotional, and social wellbeing among other things, can be impacted after transplant. However, the ways that transplant recipients experience health-related quality of life are not fully understood. The aim of this review was to identify and understand the different ways that people’s lives are affected (i.e. health-related quality of life outcomes) following transplantation. This review brings together existing studies that report people’s experiences of health-related quality of life after transplantation. We identified 93 different health-related quality of life outcomes that people can experience after transplant. This included positive and negative emotions (e.g. appreciation of life, fear of rejection), social challenges (e.g. social restrictions due to risk of infection) and problems related to transplant medicine (e.g. feeling sick, weight gain). These outcomes were commonly reported across different organs (kidney, liver, heart, lung), suggesting there are some similarities in how health-related quality of life is experienced by different transplant recipients. These findings can be used to develop a survey for transplant recipients that would be able to measure the different ways that their life may be affected after transplant. The survey can then be used to help identify what supports people need to live well with their transplant.

**Supplementary Information:**

The online version contains supplementary material available at https://doi.org/10.1186/s41687-026-01158-2.

## Introduction

In 2023, 172,409 people received a solid organ transplant, worldwide [1]. Following transplantation, recipients may live for long periods. For example, 95% of liver transplant recipients are still alive at one-year post-transplant and 81% at 10-years; comparable figures for heart transplant recipients are 84% and 61% [2]. However, it is not only quantity of survival that is important, but also the quality of that survival. This may be represented through an individual’s health-related quality of life (HRQoL), which can be defined as: “*the state of wellbeing that is a composite of two components: the ability to perform everyday activities that reflect physical*,* psychological*,* and social well-being; and patient satisfaction with levels of functioning and control of the disease*” [3]. It is, therefore, important to understand what HRQoL means to solid organ transplant recipients, following transplantation.

Although transplant recipients may experience improvements in HRQoL, compared to pre-transplant [4], systematic reviews of quantitative research suggest that HRQoL among recipients is poor across several dimensions, in comparison to general population controls [5, 6]. Transplant recipients may experience psychological distress (e.g. concerns about future health) [7, 8], physical changes (e.g. weight gain) [9], social (e.g. social restrictions due to risk of infection) and role challenges (e.g. inability to work) [10]. The extent of this impact may be influenced, or vary, by the solid organ received [11] (e.g. pulmonary symptoms for lung patients [12]; nausea or appetite loss for kidney patients [13]), side-effects of immunosuppressant medications [14], or sociodemographic factors (e.g. age, sex) [15].

In transplant research, HRQoL is most commonly measured using generic patient-reported outcome measures (PROMs), such as SF-36 [16, 17]. However, generic measures may not capture all the HRQoL outcomes relevant to solid organ transplant recipients. When selecting a PROM for use in research or clinical practice, it is crucial to consider whether it has good content validity, with content (i.e. the areas/topics covered by the measure) that is relevant and meaningful to the target population [18]. A recent systematic review identified 26 condition- and transplant-specific HRQoL PROMs validated in solid organ transplant recipients [19]. While these measures are more likely to capture relevant aspects of HRQoL, the review found that those published to date have poor content validity due to insufficient patient involvement in their development, and demonstrated the need for new or “improved” measures. This, in turn, emphasises the importance of, and need for, a comprehensive understanding of qualitative evidence on the HRQoL outcomes experienced by transplant recipients [20]. In particular, a qualitative synthesis could underpin the content validity of future new, or improved, PROM(s) for solid organ transplant recipients by helping to ensure that the content comprehensively captures the HRQoL outcomes that are relevant to the experiences of that population. This systematic review of qualitative research aimed to identify and understand the HRQoL outcomes experienced post-transplant by solid organ transplant recipients.

## Methods

This systematic review of qualitative research was registered with the Prospective Register for Systematic Reviews (PROSPERO) (CRD42024541448 *anonymised for peer review*) and reported in accordance with the Enhancing Transparency in Reporting the Synthesis of Qualitative Research (ENTREQ) framework [21] (Supplementary file_ENTREQ). It addressed the following primary question: What are the HRQoL outcomes experienced post-transplant by adult solid organ transplant recipients and how are they experienced? There was also a secondary question, concerning: What are the commonalities and differences in the HRQoL outcomes experienced across different solid organs?

The SPIDER framework [22] was used to inform the search strategy and eligibility criteria: Sample (adult solid organ transplant recipients); Phenomenon of Interest (HRQoL); Design (qualitative methods, e.g. interviews, focus groups); Evaluation (lived experiences); Research type (qualitative research).

### Search strategy

Searches were conducted from inception to 17th May 2024 on five bibliographic databases: MEDLINE (OVID), Embase (OVID), CINAHL (EBSCO), PsycINFO (OVID), and Scopus. These were chosen to capture literature from multiple disciplines (e.g. medicine, allied health, psychology, sociology). The search strategy encompassed three key areas: organ transplant, HRQoL, qualitative methods (Supplementary Table 1). Previously published search strategies for solid organ transplant recipients on lived experiences in other contexts (e.g. medicine-taking) [14, 23], and quantitative reviews of HRQoL [6, 24], informed the development of search terms. To formulate a combination of appropriate medical subject headings and keywords, we consulted an experienced information specialist. Final search terms were tailored in line with the specific subject headings used by each database (Supplementary Table 2). The searches intended to identify all available studies; to retrieve any additional papers for inclusion, we hand-searched the reference lists and forward citations of eligible papers and relevant systematic reviews (e.g [23, 25]).

### Eligibility criteria

A paper was eligible for inclusion if: (1) it was an original article reporting novel data analysis from primary research, available in English; (2) it reported the findings of qualitative research; (3) it elicited the experiences (i.e. direct, first-hand accounts) of people who received a solid organ (heart, lung, liver, kidney, pancreas, intestine) transplant as an adult (aged ≥ 18 years); (4) it reported on overall HRQoL or (combinations of) single dimensions of HRQoL, such as emotional functioning or physical functioning.

A paper was excluded if: (1) it only reported the findings of quantitative research; (2) the primary focus of the study was stated to be another construct, rather than HRQoL (e.g. self-management, healthcare experiences); (3) it elicited the experiences of paediatric transplant recipients (aged < 18 years) or adults who received their transplant as a paediatric patient. Paediatric recipients are more likely to experience disruptions to key development milestones (e.g. psychosocial, educational), which could have long-term effects into adulthood (e.g. employment) [26, 27]; these are important but were deemed beyond the scope of the current work; (4) transplant recipients’ experiences were described by someone else (e.g. family-member; healthcare professional); (5) the participants had not yet received a transplant; (6) the experiences described were related to a specific intervention (e.g. mobile health intervention).

To ensure the review remained explicitly focused on identifying the HRQoL outcomes that were relevant to the *post-transplant* experiences of transplant recipients, we also excluded: (1) studies where the sample included other groups (e.g. transplant donors, candidates, family-members, healthcare professionals); and (2) studies that set out to compare pre- and post-transplant experiences. This means that studies which explicitly aimed to explore post-transplant experiences but reported minimal pre-transplant experiences to help ‘set the scene’, were considered eligible.

### Paper selection

Paper selection followed a two-stage process. Firstly, following de-duplication, titles and abstracts were independently double screened by three blinded reviewers (BR 100%; SR and SD 50% each), using Rayyan software [28]. Any citation referring to the experiences of (an aspect of) HRQoL in adult solid organ transplant recipients was taken forward for full-text screening. Full-texts were sourced online or through an inter-library loan.

**Fig. 1:**
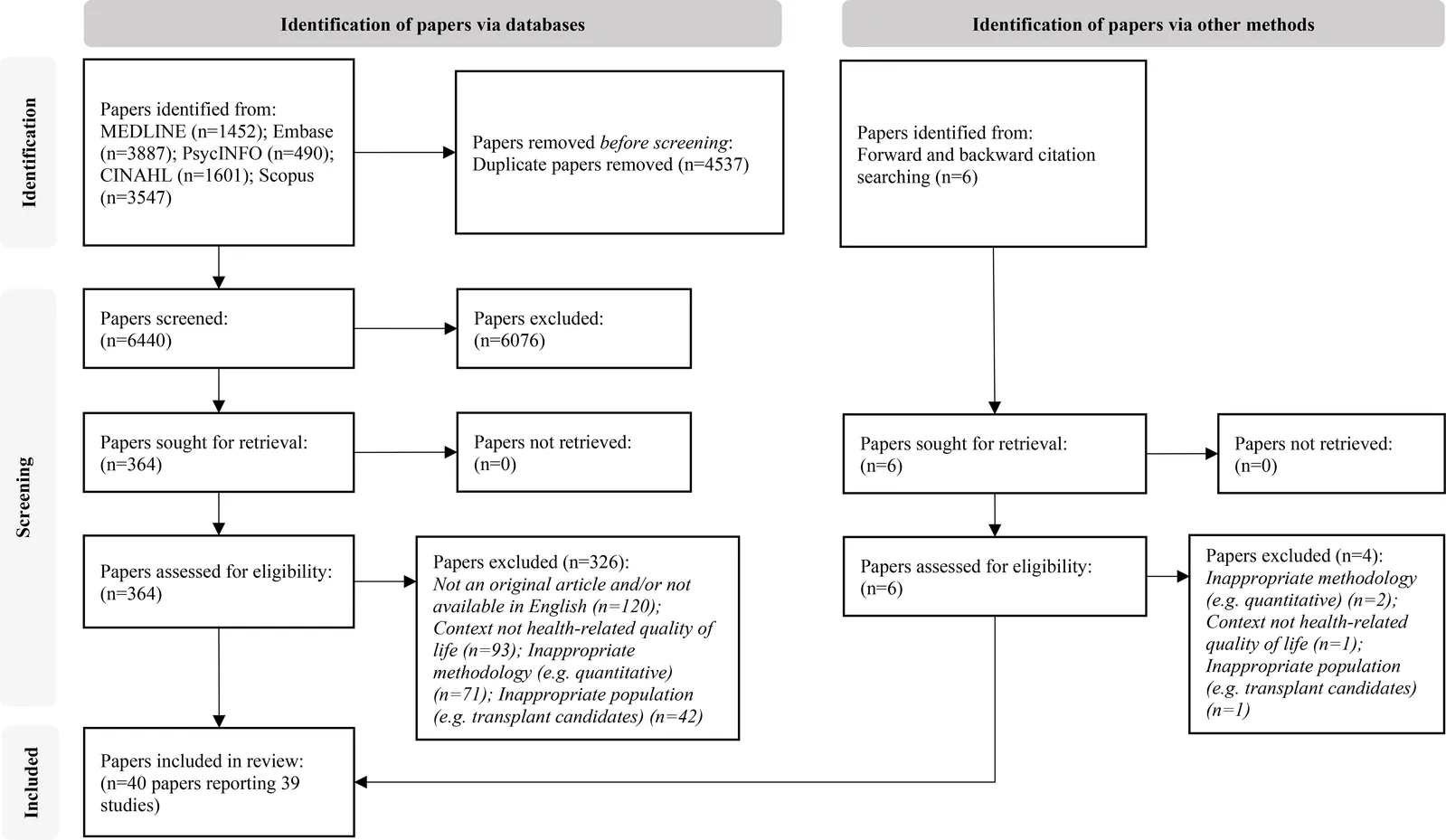
PRISMA flow diagram of paper selection

Three blinded reviewers (BR 100%; SR and SD 50% each) then double-screened the full-texts against the eligibility criteria. Papers excluded during full-text screening were given a coded exclusion reason to ensure transparency in the Preferred Reporting Items for Systematic Reviews and Meta-Analysis (PRISMA) flow diagram [29] (Fig. 1). A hierarchy of exclusion reasons was used to support consistency.

### Data extraction

Data extraction was conducted by BR and checked for accuracy by SD and SR; to facilitate this, we created, piloted, and refined a structured form. Where multiple included papers reported different findings from the same study (sample), this was treated as a single study (sample) in the synthesis. Data extraction decisions were informed by the available published and supplementary material.

We extracted the following data: study characteristics (e.g. country; aim; study design/orientation; sampling method; data collection method; data analysis strategy); sample characteristics (e.g. sample size; organ(s) received; time since transplant; socio-demographics); and author-reported key findings (e.g. themes, categories). Sample characteristics were tabulated and summarised to document the socio-demographic diversity within, and across, the samples of included studies.

### Appraisal of transparency of reporting

To appraise the reporting of each included paper, we used an amended 17-item version of the widely used Consolidated Criteria for Reporting Qualitative Health Research (COREQ) checklist [30] (Supplementary Table 3). The original 32-item COREQ checklist has been critiqued for not being generic and contributing to methodologically incongruent reporting [31]. Due to this, the review team discussed each item and reached consensus on the removal of 19 items (e.g. ‘Relationship established’, ‘Repeat interviews’, ‘Derivation of themes’), addition of four new items (e.g. ‘Approach to analysis’), and amendment of two items (e.g. ‘Data saturation’ to also consider data sufficiency). Response options were ‘Yes’ or ‘No’ for whether an item was reported; appraisal was completed by BR and checked by SD. We counted the number of included papers that reported each item, and the number of items reported by each included paper. We were conscious of the potentially reductionist nature of checklists for qualitative research, including concerns regarding their credibility [32]. Thus, appraisal was not conducted to “measure” study quality, rather highlight where there was variability, and potential room for improvement, in the reporting of qualitative research on this topic.

### Data synthesis

In accordance with the RETREAT framework for selecting a qualitative evidence synthesis approach [33], the review question, timeframe, resources, expertise, purpose, and type of data warranted the use of ‘best fit framework synthesis’ [34, 35] (Supplementary Table 4). The synthesis framework comprised nine HRQoL dimensions previously used in a systematic review of transplant-specific HRQoL PROMs [19]; these were: ‘Physical functioning’; ‘Cognitive functioning’; ‘Emotional functioning’; ‘Social functioning’; ‘Role functioning’; ‘Sexual functioning’; ‘Symptom distress’; ‘Fatigue’; and ‘Sleep’ (See Supplementary Table 5 for definitions).

Firstly, the results text and participant quotations from each included paper were imported into NVivo for storing, coding, and searching of the qualitative data. To ensure rigour and consistency, BR and two additional reviewers (SR, SD; *n* = 5 papers each) then independently conducted line-by-line coding and inductively identified the HRQoL outcomes experienced after transplant by solid organ transplant recipients. Following this, the review team discussed and reached consensus on what to label each of the identified HRQoL outcomes, and how each outcome deductively mapped to the nine dimension framework. For example, fear of graft rejection (outcome) mapped to emotional functioning (dimension). Where reviewers had conflicting interpretations of an outcome, the qualitative data was discussed. If agreement could not be reached, an additional reviewer was consulted. Throughout data synthesis we remained open to the possibility of adding more HRQoL dimensions to this framework if anything was reported in the papers that did not fit within the pre-defined dimensions; in fact, no new HRQoL dimensions were added. Following data synthesis, we counted how many included papers reported findings related to each HRQoL dimension and outcome, and the number of HRQoL dimensions and outcomes reported within the findings of each included paper. It is important to note that the number of studies reporting a given outcome is not intended to reflect its importance or prevalence among transplant recipients; rather to be transparent about aspects of HRQoL where data is plentiful or limited. Finally, we synthesised cross-cutting patterns within the HRQoL findings across the relevant studies to provide understanding of how people experienced each HRQoL dimension. Throughout paper selection, data extraction, appraisal of transparency of reporting, and data synthesis, the reviewers resolved any disagreements through discussion, involving an additional reviewer where necessary.

The findings are reported in four key areas: study characteristics, sample characteristics, findings in relation to HRQoL dimensions, and appraisal of transparency of reporting. Included papers are categorised in the tables by the relevant solid organ(s) to facilitate consideration of the secondary aim.

## Results

### Search results

We identified 10,977 hits from the database searches; following de-duplication, we screened 6440 titles and abstracts. The full texts of 364 papers were assessed for eligibility, of which 38 papers were eligible. We identified an additional six papers through hand searches, of which two were eligible. Overall, we included 40 papers reporting 39 studies (samples) in this review [36–75] (Fig. 1).

### Study characteristics

Studies were conducted in 19 countries (Table 1): eight in United Kingdom [39, 43, 44, 53, 56, 57, 64, 72], six in Iran [58, 61, 62, 65, 66, 75], four in Sweden [54, 60, 73, 74], three in Denmark [41, 68, 71], two each in Brazil [37, 47], Switzerland [52, 69], and Turkey [49, 63], and one each in Australia [46], China [42], Ecuador [36], France [50], India [38], Netherlands [48], New Zealand [59], Norway [55], Pakistan [40], Republic of Korea [67], Romania [45], and United States of America [51]. Study design/orientation was reported by 27 (of 39) studies [36, 38, 39, 41, 43, 45, 47, 49, 50, 52–57, 59–61, 63, 64, 66, 67, 69, 71, 73–75], most commonly: hermeneutic phenomenology (*n* = 6) [36, 41, 55, 60, 66, 75]; phenomenology (*n* = 6) [38, 53, 54, 56, 57, 67]; grounded theory (*n* = 4) [39, 61, 64, 73]; and descriptive (*n* = 4) [47, 51, 59, 71]. All studies, except one [69], were cross-sectional. Sampling method was reported by 25 (of 39) studies [36, 38, 39, 42, 43, 45, 46, 48, 49, 52, 53, 56–58, 61–68, 71, 73, 75], and was predominantly stated to be purposive sampling (*n* = 17) [38, 39, 43, 45, 46, 48, 52, 53, 56–58, 61–63, 65, 66, 75]; other methods used included convenience (*n* = 3) [36, 42, 68], theoretical (*n* = 3) [61, 64, 73], and criterion sampling (*n* = 2) [49, 63]. Most studies recruited participants through hospitals (*n* = 33) [36–41, 43, 44, 46, 47, 49, 50, 52–69, 71–73]. Other studies recruited through community organisations (*n* = 4) [42, 45, 48, 51], or both hospital and community (*n* = 1) [75]; recruitment pathway was not reported for one study [74].

**Table 1:** Study characteristics

Study (Country)	Aim	Study design/ orientation	Sampling method†	Recruitment‡	Data collection method	Location of data collection	Data analysis strategy
*Kidney recipients*							
Abarca-Durán (2021) [36] (Ecuador)	To explore and understand the experiences and perspectives of kidney transplant recipients regarding their sexuality.	Hermeneutic phenomenology	Convenience	Hospital	Focus groups (*n* = 9), In-depth interviews (*n* = 9)	Face-to-face	Hermeneutic phenomenology (Gadamerian)
de Brito (2015) [37] (Brazil)	To identify the main gains and stressors perceived by the patient one year subsequent to kidney transplantation.	NR	NR	Hospital	Semi-structured interviews	Face-to-face	Discourse of the Collective Subject approach
Dutta (2023) [38] (India)	To explore the lived experiences of post-kidney transplant patients.	Phenomenology	Purposive	Hospital (Department of renal transplant surgery)	In-depth interviews	NR	Colaizzi’s phenomenological data analysis
Jones (2020) [39] (UK)	To explore why transplant patients experience unexpected mild-to-moderate distress and what support they may need.	Grounded theory	Purposive	Hospital	Interviews	Face-to-face, Remote	Grounded theory, Thematic analysis
Kamran (2016) [40] (Pakistan)	To explore transplant recipients’ perceptions and transitions concerning their personality and life orientation as a consequence of a major surgical experience.	NR	NR	Hospital (Private renal clinics)	Semi-structured interviews	NR	Thematic analysis
Kristensen (2021) [41] (Denmark)	To investigate patients’ existential experiences in everyday life six months after a kidney transplantation with a living donor.	Hermeneutic phenomenology	NR	Hospital (Kidney transplant centres)	Interviews	Face-to-face	Structural analysis
Luk (2004) [42] (China)	To explore the health-related quality of life of renal transplant patients.	NR	Convenience	Community (Alliance for Renal Patients Mutual Help Association)	Interviews	NR	Qualitative content analysis
Ó Lonargáin (2017) [43] (UK)	To explore the psychological experiences of receiving a kidney transplant from a deceased donor.	Interpretative phenomenological analysis	Purposive	Hospital (NHS renal service)	Semi-structured interviews	Face-to-face	Interpretative phenomenological analysis
Orr (2007) [44] (UK)	To explore the experience of living with a transplanted kidney, in order to gain a deeper understanding of its impact on recipients’ quality of life as they see it.	NR	NR	Hospital (Renal unit)	Focus groups	NR	Thematic analysis
Petre (2021) [45] (Romania)	To explore how individuals experience life with a transplanted kidney from a deceased donor.	Interpretative phenomenological analysis	Purposive	Community (Romanian Transplant Recipients Association)	Semi-structured interviews	Face-to-face	Interpretative phenomenological analysis
Pinter (2017) [46] (Australia)	To describe the range and depth of the experiences and perspectives of older kidney transplant recipients about their expectations and treatment goals while living and aging with a kidney transplant.	NR	Purposive	Hospital (Dialysis units)	Semi-structured interviews	NR	Thematic analysis
Santos (2016) [47] (Brazil)	To know the advantages and difficulties that people with chronic kidney disease experience after renal transplantation.	Descriptive, Critical Incident Technique	NR	Hospital (Nephrology services)	Semi-structured interviews	Face-to-face	Critical Incident Technique
Schipper (2014) [48] (Netherlands)	To describe the wide range of issues that patients may experience after transplantation, as well as the strategies they use to adapt to the transplantation.	NR	Purposive	Community (Online patient organisation)	Interviews (*n* = 18), Focus groups (*n* = 12	Face-to-face	Inductive content analysis
Tasdemir (2023) [49] (Turkey)	To explore the long-term problems and experiences of adults who have had a kidney transplant due to end-stage kidney disease after discharge.	Descriptive phenomenology	Criterion	Hospital (Organ transplant centre polyclinic)	Semi-structured interviews	Face-to-face	Colaizzi’s phenomenological data analysis
*Liver recipients*							
Bardet (2014) [50] (France)	To gain detailed insight concerning liver transplanted patients’ personal beliefs about liver transplantation, graft rejection, and immunosuppressant medications.	Common sense model	Unclear	Hospital (Liver transplant centre)	Interviews	Face-to-face	NR
Bean (2005) [51] (USA)	To determine how adult liver transplant recipients describe their quality of life.	Descriptive	NR	Community (In-person support group)	Interviews	Face-to-face	Basic descriptive analysis
Beckmann (2022) [52] (Switzerland)	To explore patients’ experiences with gaining weight after liver transplantation.	NR	Purposive	Hospital	Interviews	Face-to-face	Reflexive thematic analysis
Dudley (2007) [53] (UK)	To identify factors that could impact on quality of life after liver transplantation for hepatitis C virus infection.	Phenomenology	Purposive	Hospital	In-depth interview	Face-to-face	Colaizzi’s phenomenological data analysis
Forsberg (2000) [54] (Sweden)	To search for subjective experiences of the meaning of being liver transplanted, one year after the transplantation.	Phenomenology	Unclear	Hospital	Interviews	Face-to-face	Karlsson’s phenomenological data analysis
Naden (2023) [55] (Norway)	To explore individuals’ mental, emotional and existential experiences living with a liver transplant during a period of approximately 10 years.	Hermeneutic phenomenology	NR	Hospital	Semi-structured interviews	Face-to-face	NR
Robertson (1999) [56] (UK)	To explore individuals’ perceptions of the effect of a liver transplant on their quality of life, focusing on the progression from dependence to independence physically, socially and psychologically.	Phenomenology	Purposive	Hospital	Semi-structured interviews	Face-to-face	Cluster analysis
Sargent (2007) [57] (UK)	To document the health-related quality of life in patients who have received an emergency liver transplant for acute liver failure.	Phenomenology	Purposive	Hospital	In-depth interviews	Face-to-face, Remote	Giorgi’s phenomenological data analysis
Taher (2021) [58] (Iran)	To explore patients’ experiences of post-liver transplantation life challenges.	NR	Purposive	Hospital (Liver transplant clinic)	Semi-structured interviews	NR	Qualitative content analysis
Wainwright (2018) [59] (New Zealand)	To explore liver transplant recipients’ perceptions and experiences after the first three years, including how they re-establish function in everyday life as they adapt to their new normal to achieve quality of life.	Descriptive	NR	Hospital (Liver transplant unit)	Interview	NR	Inductive thematic analysis, Constant comparison analysis
*Heart recipients*							
Almgren (2017) [60] (Sweden)	To explore the meaning of uncertainty during the first year after a heart transplantation.	Hermeneutic phenomenology	NR	Hospital	Interviews	Face-to-face	Hermeneutic phenomenology (Lindseth and Norberg)
Bahramnezhad (2022) [61] (Iran)	To explore the integration process with a donated heart in heart transplant patients.	Grounded theory	Purposive, Theoretical	Hospital (Heart transplant clinic)	Semi-structured interviews	Face-to-face	Grounded theory
Dehghan-Nayeri (2017) [62] (Iran)	To explore the physical and psychosocial outcomes of heart transplantation.	NR	Purposive	Hospital (Cardiac care clinic)	Semi-structured interviews	Face-to-face	Qualitative content analysis
Fatma (2021) [63] (Turkey)	To explore the experiences of heart transplant recipients during and after heart transplantation.	Descriptive phenomenology	Purposive, Criterion	Hospital (Cardiovascular surgery outpatient clinic)	Semi-structured interviews	Face-to-face	Qualitative content analysis
Kaba (2005) [64] (UK)	To explore psychological problems experienced by heart transplant recipients.	Grounded theory	Theoretical	Hospital	Unstructured in-depth interviews	Face-to-face	Constant comparison analysis
Pashaeypoor (2021) [65] (Iran)	To explain and interpret the experience of heart transplant recipients in Iran.	NR	Purposive	Hospital	Interviews	Face-to-face	Conventional content analysis
Peyrovi (2014) [66] (Iran)	To understand and gain deeper insight into the lived experience of Iranian heart transplant recipients.	Hermeneutic phenomenology	Purposive	Hospital (Heart clinic)	Interviews	Face-to-face	Diekelmann’s hermeneutical analysis
Yoo (2021) [67] (Republic of Korea)	To explore and describe the lived experience of young adult heart transplant recipients in Korea.	Phenomenology	Snowball	Hospital (Outpatient heart transplant clinic)	In-depth interviews	Face-to-face	Colaizzi’s phenomenological data analysis
*Lung recipients*							
Graarup (2017) [68] (Denmark)	To describe patients’ experiences throughout the first four months post-lung transplant.	NR	Convenience	Hospital	Semi-structured interviews	Face-to-face	Qualitative content analysis
Seiler (2016); Eichenlaub (2021) [69, 70] (Switzerland)	To gain a deeper understanding of patients’ individual concerns related to their lung transplantation within the first six months post-transplant.	Longitudinal	NR	Hospital (Transplant centre)	Semi-structured interviews	Face-to-face	Qualitative content analysis
Thomsen (2009) [71] (Denmark)	To investigate the experiences of everyday life after lung transplantation of patients with previous chronic obstructive pulmonary disease.	Descriptive	Maximum variation	Hospital	Semi-structured interviews	Face-to-face	Qualitative content analysis
*Mixed samples of organ recipients*							
Boaz (2014) [72] (UK)	To explore patients’ perceptions and experiences of ‘normality’ and the influences on this at three time points post-transplant.	NR	Unclear	Hospital (Transplant centre)	Semi-structured interviews	Face-to-face	NR
Forsberg (2016) [73] (Sweden)	To investigate the main concerns associated with social function after organ transplantation and how organ transplant recipients deal with these concerns.	Grounded theory	Theoretical	Hospital (Transplant centre)	Interviews	Face-to-face	Grounded theory
Nilsson (2008) [74] (Sweden)	To investigate perceptions of graft rejection and different methods to obtain knowledge about graft rejection among adult organ transplant recipients.	Phenomenography	Unclear	NR	Interviews	NR	NR
Sheikhalipour (2018) [75] (Iran)	To explore the experiences of organ recipients in the post-transplantation period.	Hermeneutic phenomenology	Purposive	Hospital (Heart clinic, Kidney transplantation ward); Community (Association of Liver Transplantation)	Semi-structured interviews	Face-to-face	Diekelmann’s hermeneutical analysis

NR = Not reported; UK = United Kingdom; USA = United States of America

†Where ‘Sampling method’ is reported as unclear, the study authors reported attempts to achieve variation in their sample but were not explicit about the method used

‡Where available, specific details for ‘Recruitment’ location is provided in parentheses

Data collection was reported by most studies to be through individual interviews (*n* = 36 studies) [37–43, 45–47, 49–69, 71–75]; where specified, interviews were primarily reported to be semi-structured. Other studies reported conducting focus groups (*n* = 1) [44], or both focus groups and individual interviews (*n* = 2) [36, 48]. Thirty-one studies [36, 37, 39, 41, 43, 45, 47–57, 60–69, 71–73, 75] reported face-to-face data collection; two of these studies [39, 57] also reported remote (telephone) data collection. Twenty different data analysis strategies were reported by 35 studies [36–49, 51–54, 56–69, 71, 73, 75]. The most commonly reported data analysis strategies were: qualitative content analysis (*n* = 7 studies) [42, 58, 62, 63, 68, 69, 71]; Colaizzi’s phenomenological data analysis (*n* = 4) [38, 49, 53, 67]; thematic analysis (*n* = 4) [39, 40, 44, 46]; and grounded theory (*n* = 3) [39, 61, 73].

### Sample characteristics

Sample size ranged from five to 50 (Table 2). Supplementary Table 6 presents an overview of the sample characteristics; here we summarise the key characteristics. The organ(s) received were primarily kidney (*n* = 18 studies) [36–49, 72–75], liver (*n* = 13) [50–59, 73–75], and heart (*n* = 12) [60–67, 72–75]; a few studies included lung recipients (*n* = 6) [68, 69, 71–74], one [72] included simultaneous pancreas-kidney recipients, and none included intestinal transplant recipients. These numbers include, where applicable, the four studies [72–75] with mixed samples of different organ types. Time since transplant at the point of data collection ranged from two weeks to 26 years; heterogeneity was common within studies; for example, participants in the study by Pinter et al. [46] ranged from six months to 25 years since transplant. Three studies [68, 69, 73] collected data at specific times since transplant (e.g. four months). Mean age of participants was typically 40s and 50s; heterogeneity was again common within studies; for example, in Forsberg et al. [73] participants’ ages ranged from 22 to 75 years. Sex-mix ranged from 11 to 75% women. Seven (of 39) studies [39, 43, 44, 46, 51, 53, 59] reported race or ethnicity; in these studies, participants were predominantly White, with minimal representation of other racial classifications or ethnic groups in four studies [39, 46, 51, 59].

**Table 2:** Sample characteristics

Study	Sample size	Time since transplant	Age	Sex	Race/Ethnicity	Relationship status
*Kidney recipients*						
Abarca-Durán (2021) [36]	18	Mean 17.7 months	Median 39 years	Women (*n* = 10), Men (*n* = 8)	NR	Married (*n* = 10), Single (*n* = 4), Divorced (*n* = 4)
de Brito (2015) [37]	50	Median 71.8 months (range 12–230 months)	Mean 44 years (SD 12.8 years)	Women (*n* = 19), Men (*n* = 31)	NR	Stable relationship (*n* = 35), NR (*n* = 15)
Dutta (2023) [38]	18	Unclear (> 6 months)	Mean 33.2 years (range 21–49 years)	Women (*n* = 2), Men (*n* = 16)	NR	Married (*n* = 12), Single (*n* = 6)
Jones (2020) [39]	15	NR	30–39 years (*n* = 3), 40–49 (*n* = 2), 50–59 (*n* = 4), 60–69 (*n* = 4), ≥ 70 (*n* = 2)	Women (*n* = 8), Men (*n* = 7)	White (*n* = 9), Indian (*n* = 4), Caribbean (*n* = 2)	NR
Kamran (2016) [40]	14	Mean 3 years (range 1–5 years)	Mean 36.9 years (range 26–47 years)	Women (*n* = 6), Men (*n* = 8)	NR	Married (*n* = 7), Divorced (*n* = 3), Single (*n* = 3), Separated (*n* = 1)
Kristensen (2021) [41]	11	Unclear (approx. 6 months)	Mean 42.8 years (range 20–59 years)	Women (*n* = 5), Men (*n* = 6)	NR	NR
Luk (2004) [42]	31	Mean 5.5 years (range 1 month − 20 years)	Mean 42.9 years (range 27–55 years)	Women (*n* = 15), Men (*n* = 16)	NR	NR
Ó Lonargáin (2017) [43]	6	Mean 13.1 months (range 7–21 months)	Mean 45 years (range 26–68 years)	Women (*n* = 1), Men (*n* = 5)	White British (*n* = 6)	Living with a partner/spouse (*n* = 4), Not living with a partner/spouse (*n* = 2)
Orr (2007) [44]	26	Mean 5.2 years (range 2–8 years)	Mean 53.3 years (range 24–72 years)	Women (*n* = 15), Men (*n* = 11)	White British (*n* = 26)	NR
Petre (2021) [45]	8	Mean 4.9 years (range 1-12years)	Mean 38.9 years (range 29–64 years)	Women (*n* = 3), Men (*n* = 5)	NR	Married (*n* = 3), In a relationship (*n* = 2), Single (*n* = 2), Divorced (*n* = 1)
Pinter (2017) [46]	30	Mean 6.4 years (range 0.5–25 years)	Mean 70.1 years (range 65–80 years)	Women (*n* = 15), Men (*n* = 15)	White (*n* = 15), Maltese (*n* = 7), Other (*n* = 5), Egyptian (*n* = 3)	Married (*n* = 23), Widowed (*n* = 3), Divorced (*n* = 2), Single (*n* = 2)
Santos (2016) [47]	20	Unclear (> 1 year)	NR	NR	NR	NR
Schipper (2014) [48]	30	Range 2–9 years	Range 27–70 years	Women (*n* = 14), Men (*n* = 16)	NR	NR
Tasdemir (2023) [49]	17	Mean 5.9 years (range 1–26 years)	Mean 42.4 years (range 28–60 years)	Women (*n* = 5), Men (*n* = 12)	NR	Married (*n* = 15), Unmarried (*n* = 2)
*Liver recipients*						
Bardet (2014) [50]	8	Unclear (> 12 months)	Mean 55.1 years (range 32–70 years)	Women (*n* = 2), Men (*n* = 6)	NR	NR
Bean (2005) [51]	12	Range 2–10 years	Range 41–68 years	Women (*n* = 7), Men (*n* = 5)	White (*n* = 11), Hispanic (*n* = 1)	Married (*n* = 11), Single (*n* = 1)
Beckmann (2022) [52]	12	Median 23 months (range 17–58 months)	Mean 56.8 years (range 23–72 years)	Women (*n* = 7), Men (*n* = 5)	NR	NR
Dudley (2007) [53]	8	Median 5.5 years (range 3–7 years)	Median 51 years (range 44–60 years)	Women (*n* = 2), Men (*n* = 6)	Caucasian (*n* = 8)	Married (*n* = 7), Widowed (*n* = 1)
Forsberg (2000) [54]	12	Unclear (> 12 months)	Mean 51 years (range 24–63 years)	Women (*n* = 9), Men (*n* = 3)	NR	Married (*n* = 7), Divorced (*n* = 2), Single (*n* = 2), Widowed (*n* = 1)
Naden (2023) [55]	9	Unclear (approx. 10 years)	Mean 54 years (range 38–67 years)	Women (*n* = 2), Men (*n* = 7)	NR	NR
Robertson (1999) [56]	5	Unclear (> 1 year)	NR	NR	NR	NR
Sargent (2007) [57]	6	Mean 3 years 1 month (range 1 year 6 months − 5 years 6 months)	Mean 34 years 11 months (range 18 years 6 months − 66 years 10 months)	Women (*n* = 2), Men (*n* = 4)	NR	NR
Taher (2021) [58]	18	Mean 2.4 years (range 4 months − 12 years)	Mean 51 years (range 29–63 years)	Women (*n* = 8), Men (*n* = 10)	NR	Married (*n* = 17), Single (*n* = 1)
Wainwright (2018) [59]	17	NR	NR	Women (*n* = 8), Men (*n* = 9)	New Zealand European (*n* = 11), Māori (*n* = 4), Other (*n* = 2)	NR
*Heart recipients*						
Almgren (2017) [60]	14	Unclear (< 12 months)	Mean 51 years (range 28–67 years)	Women (*n* = 4), Men (*n* = 10)	NR	NR
Bahramnezhad (2022) [61]	15	Mean 4 years (SD 1.05 years)	Mean 48.5 years (SD 3.09 years)	Women (*n* = 9), Men (*n* = 6)	NR	NR
Dehghan-Nayeri (2017) [62]	15	Mean 19.5 months	Mean 43.4 years	Women (*n* = 2), Men (*n* = 13)	NR	NR
Fatma (2021) [63]	11	Mean 53.5 months (range 2–10 years)	Mean 44 years (range 29–57 years)	Women (*n* = 2), Men (*n* = 9)	NR	Married (*n* = 11)
Kaba (2005) [64]	42	Range 2–24 months	Range 32–61 years	Women (*n* = 7), Men (*n* = 35)	NR	NR
Pashaeypoor (2021) [65]	13	Mean 5.9 years (range 1–11 years)	Mean 40.5 years (range 23–60 years)	Women (*n* = 6), Men (*n* = 7)	NR	Married (*n* = 9), Single (*n* = 4)
Peyrovi (2014) [66]	11	Median 48 months (range 7-216 months)	Median 30 years (range 21–55 years)	Women (*n* = 2), Men (*n* = 9)	NR	Married (*n* = 5), Single (*n* = 6)
Yoo (2021) [67]	15	Mean 7.1 years (range 1.6–13.7 years)	Mean 31.3 years (range 25–34 years)	Women (*n* = 7), Men (*n* = 8)	NR	Married (*n* = 4), Single (*n* = 11)
*Lung recipients*						
Graarup (2017) [68]†	26	4 months	Mean 50.7 years (range 26–61 years)	Women (*n* = 11), Men (*n* = 15)	NR	Living with a partner (*n* = 17), NR (*n* = 14)
Seiler (2016); Eichenlaub (2021) [69, 70]‡	40	2 weeks, 3 months, 6 months	Median 50 years (range 20–68 years)	Women (*n* = 18), Men (*n* = 22)	NR	Married (*n* = 17), Single (*n* = 14), Divorced (*n* = 6), Widowed (*n* = 3)
Thomsen (2009) [71]	10	Range 7.5–86 months	Range 51–69 years	Women (*n* = 5), Men (*n* = 5)	NR	Married (*n* = 4), Live alone (*n* = 6)
*Mixed samples of organ recipients*						
Boaz (2014) [72]	25: Kidney (*n* = 20, *n* = 2 with previous Heart/Lung), SPK (*n* = 5)	Range 3 months − 25 years	Mean 47 years (range 19–65 years)	Women (*n* = 10), Men (*n* = 15)	NR	NR
Forsberg (2016) [73]	20: Heart (*n* = 6), Lung (*n* = 4), Liver (*n* = 4), Kidney (*n* = 5), Liver/Kidney (*n* = 1)	1 year	Mean 54.4 years (range 22–75 years)	Women (*n* = 7), Men (*n* = 13)	NR	NR
Nilsson (2008) [74]	16: Lung (*n* = 4), Kidney (*n* = 4), Liver (*n* = 4), Heart (*n* = 3), Heart/Kidney (*n* = 1)	Mean 3.6 years (range 3 months − 10 years)	Mean 47.4 years (range 21–63 years)	Women (*n* = 10), Men (*n* = 6)	NR	NR
Sheikhalipour (2018) [75]	15: Heart (*n* = 8), Kidney (*n* = 4), Liver (*n* = 3)	Mean 3.8 years (range 9 months − 12 years)	Mean 45.1 years (range 27–68 years)	Women (*n* = 7), Men (*n* = 8)	NR	Married (*n* = 14), Single (*n* = 1)

NR = Not reported; SD = Standard deviation; SPK = Simultaneous Pancreas-Kidney

†With the exception of ‘Sex’, Graarup (2017) reported sample characteristics for *n* = 31 lung recipients, but only interviewed *n* = 26, so age and relationship status may not be accurate

‡Eichenlaub (2021) is a secondary analysis of the data collected for Seiler (2016), but did not include two of the participants. Where available, the sample characteristics reported here are for the full dataset (*n* = 40)

Eighteen (of 39) studies [38, 40, 42, 43, 45, 46, 50, 51, 53, 54, 58, 63, 65, 67, 68, 71, 73, 75] reported employment status (Supplementary Table 7). Participants were predominantly employed or retired; only four studies [45, 50, 51, 54] were explicit about whether people were unemployed or retired due to being unable to work. Six studies [37, 38, 46, 58, 59, 75] reported rurality. In these six, participants primarily lived in urban areas and only three [38, 46, 58] reported including people living in rural areas. Finally, only four studies [38, 46, 52, 53] reported whether participants had co-morbid conditions, spanning a broad range of conditions, such as diabetes and hypertension.

### Findings in relation to HRQoL dimensions

Key findings as reported by paper authors are outlined in Supplementary Table 8. Following the best fit framework synthesis, 93 HRQoL outcomes were identified (mean 24.45, SD 9.92, median 22.5, IQR 18.25-30 outcomes reported by each paper); these outcomes mapped to all nine HRQoL dimensions (mean 5.2, SD 1.49, median 5, IQR 4–6 dimensions reported by each paper) (Table 3; Supplementary Tables 5 and 9). Across all solid organs, 85 of the 93 outcomes were reported by two or more organ types; 68 by three or more organ types; and 40 by kidney, liver, heart, and lung transplant recipients (Supplementary file_Outcome map). Qualitative findings in relation to each HRQoL dimension are reported below, with supporting quotes in Supplementary Table 10. When interrelationships between dimensions were identified in the synthesis, these have been made clear in the reporting of the results.

**Table 3:** HRQoL outcomes in relation to HRQoL dimensions

HRQoL dimensions	HRQoL outcomes	Included papers with related findings
***Physical functioning***		***n* = 19** [37, 38, 42, 43, 46, 47, 52, 53, 55, 57, 61, 62, 68, 69, 71–75]
	• Physical (in)activity/fitness	*n* = 14 [38, 42, 46, 47, 52, 53, 55, 57, 68, 69, 71–73, 75]
	• Physical capability	*n* = 10 [37, 42, 46, 47, 52, 57, 62, 68, 69, 71]
	• Physical strength	*n* = 13 [38, 43, 46, 47, 52, 53, 55, 57, 61, 68, 69, 74, 75]
***Cognitive functioning***		***n* = 5** [42, 51, 52, 56, 67]
	• Concentration	*n* = 2 [52, 67]
	• Memory impairment	*n* = 3 [42, 51, 56]
***Emotional functioning***		***n* = 40** [36–75]
	• Altered sense of body	*n* = 18 [39, 40, 43–46, 48, 50, 54–56, 61, 64, 65, 68–70, 75]
	• Appreciation of life	*n* = 17 [40, 43–46, 51–53, 55–57, 59, 66, 67, 71, 73, 75]
	• Body image	*n* = 13 [36, 37, 39–41, 45, 48, 52, 57, 67, 69, 71, 72]
	• Consideration of the donor (family)	*n* = 21 [38–41, 43–46, 49, 53, 54, 56, 59, 61, 62, 64–66, 69–71]
	• Distress	*n* = 10 [37, 39, 43, 45, 52, 58, 61, 66, 69, 71]
	• Emotional preparedness	*n* = 12 [39, 44–46, 48, 50, 53, 56, 60, 68, 72, 75]
	• Facing death/trauma	*n* = 15 [45, 46, 49–52, 54, 57, 59, 61, 63, 65–67, 75]
	• Fear of cancer	*n* = 3 [43, 45, 46]
	• Fear of dialysis	*n* = 9 [37, 40, 43–46, 49, 50, 72]
	• Fear of disease recurrence	*n* = 4 [53, 58, 61, 62]
	• Fear of graft rejection/failure	*n* = 25 [37–42, 44–46, 48–51, 53, 54, 58–60, 62, 66, 69–72, 74]
	• Fear of infections	*n* = 9 [45, 49, 50, 57, 60, 63, 69–71]
	• Fear of medication side-effects	*n* = 5 [40, 45, 46, 50, 60]
	• Fear of missed medication	*n* = 3 [46, 47, 50]
	• Feeling vulnerable	*n* = 12 [41, 43, 45–47, 50, 55, 59, 69, 71, 72, 74]
	• Feelings of control/helplessness	*n* = 25 [37, 39–42, 44–46, 48, 50, 52, 54–56, 59, 60, 62, 66–70, 72, 74, 75]
	• Feelings of isolation	*n* = 11 [38, 39, 45, 48, 50, 54, 57, 60, 61, 69, 73]
	• Feelings of peace	*n* = 3 [51, 62, 75]
	• Future planning/changing priorities	*n* = 13 [38, 40–42, 45, 46, 48, 53, 55, 58, 59, 68, 71]
	• Future uncertainty	*n* = 25 [39–41, 43–46, 48, 51–55, 58, 60–63, 66–69, 71, 72, 74]
	• Gratitude	*n* = 25 [38, 40, 41, 43–46, 48, 50–55, 57, 59, 62–65, 67–69, 71, 73]
	• Guilt	*n* = 21 [36, 38–41, 43–45, 48, 53–55, 59–62, 64, 65, 70, 71, 74]
	• Health perceptions	*n* = 22 [38, 40, 42, 44, 46–50, 52–55, 60, 64, 67–70, 72, 74, 75]
	• Identity/worth	*n* = 17 [39–41, 44–46, 48, 51, 54, 59, 61, 62, 65, 67–69, 74]
	• Low mood	*n* = 13 [39, 40, 43, 51, 54, 55, 58, 60, 63, 66, 67, 69, 72]
	• Mood swings	*n* = 4 [42, 54, 69, 72]
	• New possibilities	*n* = 13 [37, 41, 43, 47, 50, 51, 58, 66–69, 72, 75]
	• Optimism/hope	*n* = 15 [39–41, 43, 45, 46, 53, 55, 57, 58, 66, 68, 69, 71, 75]
	• Outlook on life	*n* = 25 [39, 40, 43–49, 52–57, 59, 60, 66–72, 74]
	• Patient burden	*n* = 19 [37, 38, 40, 42–44, 46, 47, 49, 50, 55, 63, 66–72]
	• Perceptions of transplantation	*n* = 8 [37, 45–47, 49, 51, 53, 66]
	• Personality/mood changes	*n* = 8 [59, 62, 63, 65, 67, 72, 73, 75]
	• Religious/spiritual beliefs	*n* = 8 [45, 51, 61–63, 65, 66, 75]
	• Second chance	*n* = 20 [40, 41, 43, 44, 46, 48–54, 57, 59, 61, 63, 67–69, 71]
	• Self-confidence/insecurities	*n* = 9 [40, 45, 46, 57, 60, 68, 69, 71, 74]
***Social functioning***		***n* = 37** [36–54, 56–63, 66–75]
	• Finding a new partner	*n* = 6 [36, 40, 45, 66, 67, 71]
	• Interactions with other transplant recipients	*n* = 14 [39, 42–45, 50, 51, 56, 57, 59, 63, 67, 71, 74]
	• (Lack of) understanding from others†	*n* = 18 [37, 39, 41, 43–45, 48–50, 53, 56, 57, 59–61, 63, 66, 72]
	• Medication interference	*n* = 10 [38, 40, 44, 47, 49, 50, 67–69, 72]
	• Relationship with partner	*n* = 17 [36, 40, 43, 45–48, 54, 59, 60, 63, 66–68, 71–73]
	• Relationships with employer/colleagues	*n* = 5 [43–45, 59, 72]
	• Relationships with family	*n* = 24 [37, 38, 40, 42, 43, 45–47, 50–54, 59, 60, 63, 66–69, 71–73, 75]
	• Relationships with friends	*n* = 10 [38, 39, 42, 45, 54, 57, 60, 67, 71, 73]
	• Risk of infection	*n* = 13 [38, 42, 44, 45, 47, 49, 50, 57, 63, 69–71, 73]
	• Risk of skin cancer	*n* = 7 [44, 45, 47, 49, 57, 71, 72]
	• Social opportunities/restrictions	*n* = 19 [37, 38, 41, 43, 44, 46–48, 50, 54, 56, 58, 62, 63, 67, 69, 71–73]
***Role functioning***		***n* = 35** [37–62, 65–69, 71–73, 75]
	• Ability to work	*n* = 16 [40, 42, 43, 45, 47, 48, 51, 55, 58, 60, 62, 67, 68, 71–73]
	• Autonomy	*n* = 15 [37, 38, 41–48, 57, 68, 69, 71, 75]
	• Driving	*n* = 2 [47, 59]
	• Financial implications	*n* = 13 [38, 40, 42, 44, 45, 50, 51, 58, 60, 62, 66, 67, 75]
	• Finding employment/employability	*n* = 8 [37, 42, 43, 51, 61, 62, 67, 68]
	• Hobbies/leisure activities	*n* = 11 [39, 42, 43, 47, 48, 50, 51, 53, 69, 71, 72]
	• Household tasks	*n* = 4 [47, 59, 71, 72]
	• (In)dependence	*n* = 14 [37, 38, 40, 46–48, 54, 56, 57, 59, 68, 69, 71, 73]
	• Returning to ‘normal’	*n* = 20 [37, 41, 43, 45, 47, 48, 50–52, 55–59, 65, 67, 68, 71, 72, 75]
	• Returning to work/changes in employment	*n* = 16 [38, 41, 42, 45, 50–52, 57–60, 62, 67, 68, 72, 73]
	• Self-care	*n* = 4 [37, 58, 69, 71]
	• Travelling	*n* = 12 [42, 44, 46–49, 60, 68, 69, 71–73]
***Sexual functioning***		***n* = 12** [36, 40, 45, 48, 54, 58, 61, 66, 67, 71–73]
	• Attitudes towards sexual activity	*n* = 2 [36, 71]
	• Interest in sexual activity	*n* = 4 [36, 54, 71, 73]
	• Reproduction/fertility	*n* = 7 [36, 45, 58, 61, 66, 67, 72]
	• Sexual capability	*n* = 4 [36, 45, 66, 71]
	• Sexual fears/concerns‡	*n* = 4 [36, 40, 48, 54]
***Symptom distress***		***n* = 26** [36, 37, 39–48, 52–54, 57, 58, 62, 63, 66–72]
	• Breathlessness	*n* = 2 [53, 71]
	• Changes in appetite	*n* = 6 [41, 42, 46, 52, 54, 68]
	• Comorbidities	*n* = 3 [36, 46, 72]
	• Gastrointestinal symptoms	*n* = 7 [46, 57, 62, 68–70, 72]
	• Hair growth/loss	*n* = 12 [36, 39–42, 44, 45, 48, 67, 69, 71, 72]
	• Hypertension	*n* = 3 [42, 44, 69]
	• Infections	*n* = 4 [43, 46, 62, 69]
	• Musculoskeletal symptoms	*n* = 2 [62, 63]
	• Neurological symptoms	*n* = 4 [42, 63, 69, 72]
	• New onset diabetes	*n* = 5 [37, 42, 46, 69, 72]
	• Oral symptoms	*n* = 4 [42, 44, 46, 63]
	• Osteoporosis	*n* = 4 [40, 42, 44, 72]
	• Pain/discomfort	*n* = 14 [37, 42, 46, 47, 52, 54, 57, 58, 66, 68–72]
	• Scars	*n* = 4 [36, 40, 41, 67]
	• Skin symptoms	*n* = 6 [42, 44, 45, 67, 71, 72]
	• Swelling	*n* = 3 [63, 67, 69]
	• Urogenital symptoms	*n* = 1 [42]
	• Visual impairment	*n* = 1 [42]
	• Weight gain/loss	*n* = 18 [36, 39–42, 44–46, 48, 52–54, 57, 62, 68, 69, 71, 72]
***Fatigue***		***n* = 24** [37, 40, 41, 44, 46, 48, 50–57, 60, 61, 67–69, 71–75]
	• Chronic fatigue	*n* = 10 [41, 44, 52, 54, 57, 60, 67, 69, 71, 75]
	• Energy/stamina§	*n* = 15 [37, 40, 44, 46, 51, 52, 54, 55, 57, 67–69, 71, 72, 75]
	• Tiredness	*n* = 19 [41, 44, 46, 48, 50–53, 55–57, 61, 67–69, 72–75]
***Sleep***		***n* = 10** [42, 54, 55, 62, 64, 67–70, 72]
	• Hallucinations/dreams¶	*n* = 3 [68, 70, 72]
	• Sleep difficulties	*n* = 3 [54, 68, 69]
	• Sleep disturbances	*n* = 5 [42, 55, 62, 64, 67]

See Supplementary Table 5 for definitions of all health-related quality of life dimensions and outcomes, Supplementary Table 10 for example supporting quotes for all health-related quality of life outcomes, and Supplementary file_Outcome map for the breakdown of outcomes identified by organ type

†‘(Lack of) understanding from others’ was mapped to ‘Social functioning’ but could also map to ‘Emotional functioning’

‡‘Sexual fears/concerns’ was mapped to ‘Sexual functioning’ but could also map to ‘Emotional functioning’

§‘Energy/stamina’ was mapped to ‘Fatigue’ but could also map to ‘Physical functioning’

¶‘Hallucinations/dreams’ was mapped to ‘Sleep’ but could also map to ‘Cognitive functioning’

### Physical functioning

Nineteen papers [37, 38, 42, 43, 46, 47, 52, 53, 55, 57, 61, 62, 68, 69, 71–75] reported findings in relation to ‘Physical functioning’, across three HRQoL outcomes (Table 3). Transplant recipients reflected on impairments in their physical capabilities, particularly concerning mobility and flexibility; this was compounded by weight gain and feelings of pain (linking to dimension ‘Symptom distress’). For many, the muscle loss experienced in the initial hospital recovery, invoked feelings of longer-term physical weakness. While people reported gradual improvements over time, physical strength often did not return to pre-transplant levels, with implications for their ability to fulfil specific work tasks, such as heavy lifting (linking to ‘Role functioning’). Achievable levels of post-transplant physical activity (e.g. walking, cycling) varied, influenced by perceived energy levels and frailty (linking to ‘Fatigue’). Greater improvements in exercise capacity were generally reported by those who suffered with breathlessness pre-transplant.

### Cognitive functioning

Five papers [42, 51, 52, 56, 67] reported limited findings in relation to ‘Cognitive functioning’, across two HRQoL outcomes (Table 3). Where reported, some transplant recipients described cognitive challenges, specifically with memory and concentration. The authors of these papers suggested vague general implications of these challenges for daily life and occupational roles (linking to ‘Role functioning’).

### Emotional functioning

All 40 papers [36–75] reported findings in relation to ‘Emotional functioning’, across 35 HRQoL outcomes (Table 3). While people often expressed positive health perceptions and the alleviation of pre-transplant symptoms (e.g. breathlessness), many experienced an altered sense of body, with some struggling to emotionally integrate the new organ. The transplanted organ was perceived as a gift that people were grateful and relieved to have received; yet, some did not feel emotionally prepared for life post-transplant, including the ‘*burden*’ of strict medication regimens and intensive follow-up care. Also, data suggested that recipients often felt guilty that someone had died so that they could live, leading to the perception of a moral imperative to make the most of their ‘second chance’ and take care of the organ, out of respect for the donor and donor family.

Transplant recipients reported several fears, primarily, of graft rejection/failure and infections. These fears stemmed from concerns about health deterioration and were intensified in those who feared a return to dialysis. The data indicated that adherence to medication and self-care gave people feelings of control, yet the unpredictable nature of graft rejection and knowledge that grafts have a limited, uncertain life expectancy often led to feelings of helplessness and vulnerability. Due to this, people reported being conscious of ‘*living on borrowed time*’ when planning for the future, with some reevaluating their career goals.

Many transplant recipients experienced low mood and distress, and in some cases, depression. Varied changes in temperament (e.g. patience, frustration) were reported, with susceptibility to mood swings. Symptoms, such as weight changes, scars (linking to ‘Symptom distress’) had implications for people’s self-confidence and body image. Further, changes in employment (linking to ‘Role functioning’) and relationships with family and friends (linking to ‘Social functioning’) had emotional implications for people’s identity and self-worth. Such emotional impact often invoked feelings of isolation.

The data presented that the trauma of facing their mortality pre-transplant led to a renewed appreciation of life post-transplant, with feelings of peace and new possibilities (e.g. pursuing new hobbies). This was due, for example, to improvements in physical functioning and freedom from dialysis (linking to ‘Physical and Role functioning’). Some recipients expressed strengthened religious and spiritual beliefs, with transplantation perceived as a ‘blessing from God’. Despite future uncertainty, many had a positive and determined outlook on life, and expressed feelings of optimism and hope that the improvements afforded by transplantation would be sustained.

### Social functioning

Thirty-seven papers [36–54, 56–63, 66–75] reported findings in relation to ‘Social functioning’, across 11 HRQoL outcomes (Table 3). Transplant recipients reflected on the ways their relationships were impacted following the transplant, including those with their partner/spouse, family, and friends. These reflections encompassed the support they had received, perceptions of how transplantation had impacted those around them (e.g. emotional distress), and changes in dynamics, such as involvement in parenting or housework (linking to ‘Role functioning’). Some recipients experienced difficulties with maintaining relationships, especially with friends; this was compounded by a perceived lack of understanding from others around transplant-related challenges and limitations. This also extended to people’s relationships with employers and work colleagues, who recipients reported were often unsure how to treat the individual in the workplace. Thus, people favoured the shared understanding found in discussing concerns around transplantation with other transplant recipients.

Transplant recipients experienced several barriers to engaging in social activities, feeling ‘*dominated*’ by strict medication regimens and dietary restrictions, which impacted their ability to go out for a meal or take day trips, for example. The heightened risk of infection and skin cancer invoked restrictions that required strict precautions (e.g. social distancing, using sunscreen); this had extended implications for vacations and crowded social events. Overall, social restrictions and others’ understanding influenced recipients’ perceptions of their ability to develop new social connections, particularly concerning intimate relationships.

### Role functioning

Thirty-five papers [37–62, 65–69, 71–73, 75] reported findings in relation to ‘Role functioning’, across 12 HRQoL outcomes (Table 3). In the initial months following transplantation, people reported a need for assistance, before gradually achieving independence (e.g. with self-care) and regaining freedom in their daily life. This sense of freedom was particularly reported by kidney recipients who had been on dialysis pre-transplant. Transplant recipients particularly valued (re)gaining autonomy and independence with respect to successful involvement in household tasks (e.g. cooking, cleaning) and driving; though time was required to regain confidence with driving, due to fear of damaging the transplant site (linking to ‘Emotional functioning’). Some also felt able to pursue hobbies and leisure activities (e.g. gardening, playing music), which extended to participation in sporting activities for those with good physical functioning (linking to ‘Physical functioning’).

Transplant-related challenges and limitations (e.g. ‘Physical functioning’, ‘Fatigue’) had implications for occupational roles. Many recipients experienced changes in their ability to work. Though some reported improvements, others felt unable to return to work and had to negotiate changes in employment (e.g. change roles, go part-time). Where people needed to find new employment, there were concerns around employability, due to employer perceptions of health status and the demands of transplantation follow-up care. These changes in employment had financial implications; this appeared to be intensified in countries where recipients experienced out-of-pocket costs for their treatment/medical care. Financial implications extended to reports of high travel insurance costs, which alongside seasonal risks and social restrictions (linking to ‘Social functioning’), influenced whether people felt able to travel abroad. Overall, many recipients experienced a disrupted sense of normality within social and occupational roles; being able to return to activities and establish a daily routine helped people perceive a return to ‘normal’.

### Sexual functioning

Twelve papers [36, 40, 45, 48, 54, 58, 61, 66, 67, 71–73] reported findings in relation to ‘Sexual functioning’, across five HRQoL outcomes (Table 3). Transplant recipients expressed general improvements in their sexual libido, compared to pre-transplant. However, sexual functioning varied, with improvements (e.g. maintaining erections, vaginal lubrication) and problems (e.g. inability to achieve orgasm) reported. The presence of problems in sexual functioning negatively influenced people’s attitudes towards sexual activity. This was often compounded by concerns around their body image or fears that sexual activity would damage the transplant (linking to ‘Emotional functioning’). Transplantation afforded many recipients the opportunity to consider having children, though this was hampered by uncertainty about graft survival and concerns (for some) around passing on the condition that led to their transplant (linking to ‘Emotional functioning’).

### Symptom distress

Twenty-six papers [36, 37, 39–48, 52–54, 57, 58, 62, 63, 66–72] reported findings in relation to ‘Symptom distress’, across 19 HRQoL outcomes (Table 3). Transplant recipients experienced considerable physical and emotional discomfort from a wide range of symptoms, primarily attributed to side-effects of their medication. Many were related to body image (e.g. changes in weight, influenced by changes in appetite; hair growth/loss; acne; scars; swelling), and negatively impacted people’s self-confidence in social environments (linking to ‘Emotional functioning’). In addition to general pain and discomfort, potential symptoms and side-effects spanned multiple body systems (e.g. gastrointestinal; musculoskeletal; neurological; oral; respiratory). Some recipients reported developing new-onset conditions due to their medication and being immunosuppressed (e.g. diabetes; infections). Living with new conditions and other comorbidities caused further distress and created barriers to re-establishing normality (linking to ‘Role functioning’).

### Fatigue

Twenty-four papers [37, 40, 41, 44, 46, 48, 50–57, 60, 61, 67–69, 71–75] reported findings in relation to ‘Fatigue’, across three HRQoL outcomes (Table 3). Transplant recipients described and reflected on feelings of lethargy, exhaustion, and general tiredness, stressing the impact of fatigue on social and occupational roles (linking to ‘Social and Role functioning’). In some papers, authors observed that fatigue was ‘chronic’, and did not improve with rest or sleep; due to this people often perceived the need to change their future plans (e.g. career goals) (linking to ‘Emotional functioning’). Some transplant recipients reported improved energy levels compared to pre-transplant, helping them to enjoy life and time with family and friends again (linking to ‘Social functioning’). However, over time, this energy diminished for many, with implications for daily life and physical activity, such as the need for frequent breaks (linking to ‘Physical functioning’).

### Sleep

Ten papers [42, 54, 55, 62, 64, 67–70, 72] reported findings in relation to ‘Sleep’, across three HRQoL outcomes (Table 3). In the early months post-transplant, recipients sometimes experienced emotionally threatening vivid dreams and hallucinations, which they attributed to side-effects of their medication. These occurrences, alongside other fears and concerns, such as survivor’s guilt (linking to ‘Emotional functioning’), meant some found it difficult to fall sleep or get enough sleep. This was hindered still by reported sleep disturbances, due to the scheduled timing of medication, and physical side-effects, such as muscle spasms (linking to ‘Symptom distress’).

### Appraisal of transparency of reporting

Table 4 provides an overview of the appraisal of transparency of reporting, in accordance with the 17-item amended COREQ checklist (Supplementary Table 3). Transparency of reporting was adequate (mean 13.58, SD 2.21, median 13, IQR 12.75-15 reported items) across included papers (Supplementary file_COREQ). Five papers [45, 61, 63, 67, 73] reported all 17 items, while four [36, 39, 48, 49] reported 16 items and four [43, 52, 66, 75] reported 15 items. Kamran et al. [40] (eight items), Dutta et al. [38] (nine), and Nilsson et al. [74] (10) were the least transparent in their reporting.

**Table 4:** Comprehensiveness of reporting across included papers

COREQ item	Included papers reporting each item	Number of papers (%)
1. Interviewer/facilitator	[36, 37, 39, 41, 42, 44–46, 48–53, 55, 56, 58, 59, 61–63, 66–75]	31 (77.5)
2. Methodological orientation/theory	[36, 38, 39, 41, 43, 45, 47, 49–51, 53–57, 59–61, 63, 64, 66, 67, 69–71, 73–75]	28 (70)
3. Sampling	[36, 38, 39, 42, 43, 45, 46, 48, 49, 52, 53, 56–58, 61–68, 71, 73, 75]	25 (62.5)
4. Method of approach	[36, 37, 39–45, 47, 48, 50–57, 59, 61–68, 72, 73]	30 (75)
5. Sample size	[36–75]	40 (100)
6. Setting of data collection	[36, 37, 39, 41, 43, 45, 47–57, 60, 61, 63–69, 71–73, 75]	30 (75)
7. Description of sample	[36–46, 48–55, 57, 58, 60–75]	37 (92.5)
8. Interview/topic guide	[36–46, 48, 49, 51–56, 58–73, 75]	36 (90)
9. Audio/visual recording	[36, 37, 39–75]	39 (97.5)
10. Duration	[36, 37, 39, 42–49, 52–55, 58–73, 75]	32 (80)
11. Data saturation/sufficiency	[36, 38, 39, 42, 45–49, 51, 53, 54, 57, 58, 61–67, 72–74]	24 (60)
12. Approach to analysis	[36–49, 51–54, 56–71, 73, 75]	36 (87.5)
13. Details of analysis process	[36, 39, 42–46, 48–52, 54, 55, 57–63, 66–71, 73–75]	30 (75)
14. Quotations presented	[36–75]	40 (100)
15. Data identifiers†	[43–49, 52, 60, 61, 63, 65, 67, 69, 72, 73, 75]	17 (42.5)
16. Data representation†	[36, 37, 39, 41, 43–50, 52, 55, 57, 58, 60–63, 65, 67, 69, 70, 72–75]	28 (70)
17. Data and findings consistent	[36–75]	40 (100)

See Supplementary Table 3 for the complete amended COREQ checklist with guide questions, and Supplementary file_COREQ for the full appraisal of transparency of reporting

†‘Data representation’ was only not reported where there were no ‘Data identifiers’ to determine the spread of participant quotations. Those that reported ‘Data representation’ but not ‘Data identifiers’ only provided participant numbers for each quotation, without a supporting key demographic (e.g. age, sex)

## Discussion

This systematic review aimed to identify and understand the HRQoL outcomes experienced post-transplant by solid organ transplant recipients. We identified 40 papers reporting 39 qualitative studies of HRQoL in solid organ transplant recipients. Following a best fit framework synthesis, we identified and mapped 93 HRQoL outcomes to nine well-established HRQoL dimensions; the most commonly reported dimensions were emotional (*n* = 40 papers), social (*n* = 37), and role (*n* = 35) functioning. Some of the *generic* HRQoL outcomes (e.g. ‘Tiredness’, ‘Pain’, ‘Physical capability’) we identified were similar to those reported in quantitative systematic reviews of kidney [4], liver [76], heart [6], and lung transplant recipients [24]. However, most studies within such reviews used generic measures of HRQoL (e.g. SF-36); these measures may fail to capture the HRQoL outcomes that are *specific* to transplantation and medication side-effects. For example, we identified several HRQoL outcomes (e.g. ‘Guilt’; ‘Social restrictions’; ‘Reproduction/fertility’) that were not found in a scoping review of key health items in PROMs used among solid organ transplant recipients [77]. Therefore, the intended analytic contribution of our review was to generate a publicly available framework for researchers, healthcare professionals and transplant recipients to freely access, and which provides a comprehensive and consolidated understanding of the HRQoL outcomes that are experienced by transplant recipients, across every aspect of life. This provides a valuable resource, synthesising the aspects of HRQoL explored within individual studies, across an extensive body of literature. This can be subject to periodic evaluation and updates to continuously improve understanding of the relevant HRQoL outcomes. Such a framework is also of value in supporting future transplant-specific PROM development. For example, it may inform the concept elicitation phase of development, alongside interviews or focus group discussions with transplant recipients. This, in turn, will help underpin the content validity of any such PROMs [18]. This is important because a systematic review of patient perspectives on PROM use in clinical care found that people prefer PROMs that are relevant to their condition [78].

Across solid organs, there were many commonalities in the HRQoL outcomes experienced by transplant recipients; specifically, 85 of the 93 identified outcomes were reported for two or more organ types (Supplementary file_Outcome map). Discounting seven minimally reported outcomes, the only “organ-specific” outcome was ‘Fear of dialysis’ in kidney recipients. This has implications for the scope of HRQoL PROMs for solid organ transplant recipients; the possible applicability of outcomes across multiple organ types highlights the value of developing and validating transplant-specific HRQoL PROMs for use across all solid organs. This could facilitate insights into HRQoL comparisons across organs to determine whether there are notable differences in how HRQoL is impacted for each solid organ. Still, the limited representation of lung and pancreas recipients in existing evidence means the generalisability of HRQoL outcomes across all solid organs should be interpreted with caution. This warrants further qualitative research including multiple organ types to explore similarities in the HRQoL outcomes experienced across recipients of different organs within the same study, where participants are exposed to the same semi-structured interview guide.

There were several positive experiences reported across HRQoL outcomes within ‘Physical’, ‘Emotional’, ‘Social’, ‘Role’, and ‘Sexual’ functioning. Whether a HRQoL outcome was reported to be a positive or negative experience varied across individuals. For example, people may experience strengthened or weakened ‘Relationships with friends’, influenced by the perceived level of understanding from others. Thus, a transplant-specific HRQoL PROM needs to allow recipients to report both positive and negative outcomes (and/or the extent to which an outcome has been experienced) to provide a balanced view of HRQoL post-transplant.

All papers reported emotional functioning as a key aspect of transplant recipients’ experiences, with relevant (positive and negative) findings across 35 HRQoL outcomes. Positive emotional outcomes included ‘Appreciation of life’, ‘Gratitude’, and ‘New possibilities’. Still, some data suggested initial positive outcomes may diminish as time elapses; for example, new possibilities may fade if an individual becomes fatigued. This is a key example of how the heterogeneity in time since transplant within and across included studies may have influenced the reporting and interpretation of the identified HRQoL outcomes. This points to the need for a PROM to be suitable for repeat administration to capture changes in HRQoL over time. In terms of more negative emotional outcomes, we found that transplant recipients may have extensive fears (e.g. of graft rejection, dialysis, disease recurrence, infections, cancer). In a PROM, these emotional outcomes may need to be individually measured to determine the appropriate support required; for example, individuals with fear of cancer may benefit from discussions around skin surveillance. However, many existing HRQoL PROMs are somewhat reductionist, with few, often generic, items measuring emotional functioning [19]. Therefore, future development of transplant-specific HRQoL PROMs should give greater consideration to the multi-faceted emotions experienced by transplant recipients.

The majority of studies were conducted in countries with a high human development index [79], and sample diversity, particularly concerning organ type, ethnicity and rurality, was lacking or poorly reported within and across included studies. In the US, ethnicity has been found to be a significant predictor of general health, physical and role functioning in kidney transplant recipients [80]. Further, solid organ transplant recipients have reported the psychosocial and practical challenges of living rurally, including the toll of travelling for follow-up care and poor access to local transplant-specific support [81]. Therefore, further research is required to explore whether there may be additional HRQoL outcomes experienced by underserved populations within and across countries. This would serve as a basis for determining the extent to which a PROM may be appropriate for different settings.

### Strengths and limitations

This was the first review to synthesise international qualitative evidence on the post-transplant HRQoL experiences of solid organ transplant recipients. Beyond the implications for PROM development, systematic reviews of qualitative research offer comprehensive understandings of social phenomena [82]. Although we remained open to adding new HRQoL dimensions, the use of a pre-defined framework of nine dimensions may have shaped or constrained the identification or categorisation of the HRQoL outcomes. Further, HRQoL is a broad concept; though we followed a well-established definition [3], the possibility cannot be discounted that some outcomes within our synthesis could have overlap in their interpretation as a HRQoL outcome, determinant or contextual factor. Still, advancing understanding in this area can inform clinical policy, practice and service provision by highlighting potential areas for support required within post-transplant follow-up care and healthcare interventions [83].

The rigorous search process, including five databases, hand searches of reference lists and citations, and consideration of each solid organ type, was a key strength of this review. This, and the application of blinded double screening throughout, should have minimised the possibility that relevant papers have been missed. Still, we only included papers available in English. Hence, relevant findings may have been reported within papers not published in English. Our appraisal of transparency of reporting afforded a thorough understanding of how the included qualitative studies were conducted, although we did modify an existing instrument.

## Conclusion

This review generated a comprehensive framework of HRQoL outcomes to consolidate the multi-faceted impact of transplantation on HRQoL post-transplant, across every aspect of life. These findings offer valuable insights into the development of more comprehensive transplant-specific HRQoL PROMs, by understanding the HRQoL outcomes that are relevant to transplant recipients, and their possible applicability across multiple organ types. This includes consideration of positive and negative HRQoL outcomes for a balanced view of life post-transplant, particularly concerning the complex emotions experienced by transplant recipients. More immediately, these findings indicate a wide variety of areas where further support may be required following transplantation.

## Supplementary Information

Below is the link to the electronic supplementary material.

Supplementary Material 1: Supplementary file_COREQ – full appraisal of transparency of reporting using the amended COREQ checklist.

Supplementary Material 2: Supplementary file_ENTREQ – how our systematic review was reported in accordance with the ‘Enhancing transparency in reporting the synthesis of qualitative research’ statement.

Supplementary Material 3: Supplementary file_Outcome map – breakdown of how frequently the health-related quality of life outcomes were reported across each solid organ type.

Supplementary Material 4: Supplementary tables – search strategy; database searches; amended COREQ checklist; RETREAT framework; health-related quality of life dimension and outcome definitions; summary of sample characteristics; additional sample characteristics; author-reported key findings; qualitative findings mapped to health-related quality of life dimensions; health-related quality of life dimensions and outcomes with supporting quotes.

## Data Availability

The datasets used and/or analysed during the current study are available from the corresponding author on reasonable request.

## Abbreviations

HRQoL: Health-related quality of life
PROMs: Patient-Reported Outcome Measures
PROSPERO: Prospective Register for Systematic Reviews
ENTREQ: Enhancing Transparency in Reporting the Synthesis of Qualitative Research
PRISMA: Preferred Reporting Items for Systematic Reviews and Meta-Analysis
COREQ: Consolidated Criteria for Reporting Qualitative Health Research
