# Supplementary material for: Identifying and understanding the health-related quality of life outcomes experienced by solid organ transplant recipients: a systematic review of qualitative research
Source: J Patient Rep Outcomes. 2026 Jul 24;10:171. doi: 10.1186/s41687-026-01158-2 (PMC13615260; doi:10.1186/s41687-026-01158-2)
Supplement: Supplementary file 1 [file 41687_2026_1158_MOESM2_ESM.docx]

## Supplementary file_ENTREQ

This supplementary file outlines how our systematic review was reported in accordance with the ‘Enhancing transparency in reporting the synthesis of qualitative research (ENTREQ)’ statement.

**Reference:** Tong, A., Flemming, K., McInnes, E., Oliver, S., & Craig, J. (2012). Enhancing transparency in reporting the synthesis of qualitative research: ENTREQ. BMC Medical Research Methodology, 12(1), 1–8. <https://doi.org/10.1186/1471-2288-12-181/TABLES/2>

| **Item** | **Guide and description** | **Where reported** |
| --- | --- | --- |
| 1. Aim | State the research question the synthesis addresses. | Introduction, P6 |
| 2. Synthesis methodology | Identify the synthesis methodology or theoretical framework which underpins the synthesis, and describe the rationale for choice of methodology. | Methods, Data synthesis, P1; Supplementary table 4 |
| 3. Approach to searching | Indicate whether the search was pre-planned (*comprehensive search strategies to seek all available studies*) or iterative (*to seek all available concepts until theoretical saturation is achieved*). | Methods, Search strategy, P2 |
| 4. Inclusion criteria | Specify the inclusion/exclusion criteria (*e.g. in terms of population, language, year limits, type of publication, study type*). | Methods, Eligibility criteria |
| 5. Data sources | Describe the information sources used (*e.g. electronic databases (MEDLINE, EMBASE, CINAHL, PsycINFO), hand searching, reference lists*) and when the searches were conducted; provide the rationale for using the data sources. | Methods, Search strategy |
| 6. Electronic search strategy | Describe the literature search (*e.g. provide electronic search strategies with population terms, clinical or health topic terms, experiential or social phenomena related terms, filters for qualitative research, and search limits*). | Supplementary tables 1 and 2 |
| 7. Study screening methods | Describe the process of study screening and sifting (*e.g. title, abstract and full text review, number of independent reviewers who screened studies*). | Methods, Paper selection |
| 8. Study characteristics | Present the characteristics of the included studies (*e.g. year of publication, country, population, number of participants, data collection, methodology, analysis, research questions*). | Results, Study characteristics/Sample characteristics |
| 9. Study selection results | Identify the number of studies screened and provide reasons for study exclusion (*e.g. for comprehensive searching, provide numbers of studies screened and reasons for exclusion indicated in a figure/flowchart*). | Results, Search results; Figure 1 |
| 10. Rationale for appraisal | Describe the rationale and approach used to appraise the included studies or selected findings (*e.g. assessment of conduct (validity and robustness), assessment of reporting (transparency), assessment of content and utility of the findings*). | Methods, Appraisal of transparency of reporting |
| 11. Appraisal items | State the tools, frameworks and criteria used to appraise the studies or selected findings (*e.g. existing tools: CASP, QARI, COREQ; reviewer developed tools*). Describe the domains assessed (*e.g. research team, study design, data analysis and interpretations, reporting*). | Methods, Appraisal of transparency of reporting; Supplementary table 3 |
| 12. Appraisal process | Indicate whether the appraisal was conducted independently by more than one reviewer and if consensus was required. | Methods, Appraisal of transparency of reporting |
| 13. Appraisal results | Present results of the quality assessment and indicate which articles, if any, were weighted/excluded based on the assessment and give the rationale. | Results, Appraisal of transparency of reporting; Table 4; Supplementary file_COREQ |
| 14. Data extraction | Indicate which sections of the primary studies were analysed and how the data was extracted from the primary studies (*e.g. all text under the headings “results /conclusions” were extracted electronically and entered into a computer software*). | Methods, Data synthesis, P1 |
| 15. Software | State the computer software used, if any. | Methods, Data synthesis, P1 |
| 16. Number of reviewers | Identify who was involved in coding and analysis. | Methods, Data synthesis, P1 |
| 17. Coding | Describe the process for coding of data (e.g. line by line coding to search for concepts). | Methods, Data synthesis, P1 |
| 18. Study comparison | Describe how comparisons were made within and across studies (*e.g. subsequent studies were coded into pre-existing concepts, and new concepts were created when deemed necessary*). | Methods, Data synthesis, P1 |
| 19. Derivation of themes | Explain whether the process of deriving the themes or constructs was inductive or deductive. | Methods, Data synthesis, P1 |
| 20. Quotations | Provide quotations from the primary studies to illustrate themes/constructs, and identify whether the quotations were participant quotations or the author’s interpretation. | Supplementary table 10 |
| 21. Synthesis output | Present rich, compelling and useful results that go beyond a summary of the primary studies (*e.g. new interpretation, models of evidence, conceptual models, analytical framework, development of a new theory or construct*). | Results, Findings in relation to QoL dimensions; Table 3; Supplementary tables 9 and 10; Supplementary file_Outcome map |
| *P = Paragraph* | | |
