# Supplementary material for: Identifying and understanding the health-related quality of life outcomes experienced by solid organ transplant recipients: a systematic review of qualitative research
Source: J Patient Rep Outcomes. 2026 Jul 24;10:171. doi: 10.1186/s41687-026-01158-2 (PMC13615260; doi:10.1186/s41687-026-01158-2)
Supplement: Supplementary file 2 [file 41687_2026_1158_MOESM4_ESM.docx]

**Identifying and understanding the health-related quality of life outcomes experienced by solid organ transplant recipients: a systematic review of qualitative research**

Contents

[Supplementary table 1. Search strategy	2](#_Toc201586598)

[Supplementary table 2. Database searches	3](#_Toc201586599)

[Supplementary table 3. Amended COREQ checklist	9](#_Toc201586600)

[Supplementary table 4. RETREAT framework	10](#_Toc201586601)

[Supplementary table 5. Health-related quality of life dimension and outcome definitions	11](#_Toc201586602)

[Supplementary table 6. Summary of sample characteristics across all included studies	15](#_Toc201586603)

[Supplementary table 7. Additional sample characteristics	17](#_Toc201586604)

[Supplementary table 8. Author-reported key findings	20](#_Toc201586605)

[Supplementary table 9. Qualitative findings mapped to health-related quality of life dimensions	24](#_Toc201586606)

[Supplementary table 10. Health-related quality of life dimensions and outcomes with supporting quotes	26](#_Toc201586607)

## Supplementary table 1. Search strategy

| **#** | **Key area** | **Keywords** |
| --- | --- | --- |
| 1 | Organ transplant | ((organ OR heart OR cardiothoracic OR cardiac OR lung OR pulmonary OR respirat* OR liver OR kidney OR renal OR pancreas OR intestin* OR multivisceral) adj2 (transplant* OR graft* OR allograft*)) OR transplant recipients/ OR organ transplantation/ OR heart transplantation/ OR heart-lung transplantation/ OR lung transplantation/ OR liver transplantation/ OR kidney transplantation/ OR pancreas transplantation/ |
| 2 | Quality of life | quality of life/ OR “quality of life” OR emotion* OR psycholog* OR role OR physical OR social OR cognit* OR fatigue OR sleep* OR symptom* OR function* OR wellbeing OR “well-being” OR normalcy OR normality OR identity |
| 3 | Qualitative methods | qualitative research/ OR “qualitative research” OR interview/ OR interview* OR focus groups/ OR “focus group*” OR ((narrative OR “semi-structured” OR semistructured OR unstructured OR structured OR open OR “in-depth” OR indepth) adj (interview* OR discussion*)) OR phenomenolog* OR thematic OR interpretive OR qualitative OR ethnograph* OR “lived experience*” |

## Supplementary table 2. Database searches

Supplementary table 2a. MEDLINE

Supplementary table 2b. Embase

Supplementary table 2c. PsycInfo

Supplementary table 2d. CINAHL

Supplementary table 2e. Scopus

## Supplementary table 3. Amended COREQ checklist

| **Topic** | **Item no.** | **Guide questions/description** |
| --- | --- | --- |
| *Personal characteristics* | | |
| Interviewer/facilitator | 1 | Which author(s) conducted the interview or focus group? |
| *Theoretical framework* | | |
| Methodological orientation/ theory | 2 | What methodological orientation was stated to underpin the study (e.g. grounded theory, phenomenology)? |
| *Participant selection* | | |
| Sampling | 3 | How were participants selected (e.g. purposive, convenience)? |
| Method of approach | 4 | How were participants approached (e.g. face-to-face, email)? |
| Sample size | 5 | How many participants were in the study? |
| *Data collection* | | |
| Setting of data collection | 6 | Where was the data collected (e.g. home, clinic, remote)? |
| Description of sample | 7 | What are the important characteristics of the sample (e.g. demographics)? |
| Interview/topic guide** | 8 | Were topics, questions, prompts, guides provided by the authors? |
| Audio/visual recording | 9 | Did the researcher use audio or visual recording to collect the data? |
| Duration | 10 | What was the duration of the interviews or focus groups? |
| Data saturation/sufficiency** | 11 | Was data saturation/sufficiency discussed? |
| *Data analysis* | | |
| Approach to analysis* | 12 | What approach was used to analyse the data – inductive generation/deductive mapping to existing frameworks? |
| Details of analysis process* | 13 | Who was involved in the analysis? What was the analysis process? |
| *Reporting* | | |
| Quotations presented | 14 | Were participant quotations presented to illustrate the themes/findings? |
| Data identifiers* | 15 | Was each quotation identified (e.g. participant number, key demographic)? |
| Data representation* | 16 | Was there reasonable spread of participant representation in the quotations presented? |
| Data and findings consistent | 17 | Was there consistency between the data presented and the findings? |
| *New item added by the review team.  **Item wording amended from original checklist. | | |

For the full appraisal of transparency of reporting using the amended COREQ checklist, please see ‘*Supplementary file_COREQ*’.

## Supplementary table 4. RETREAT framework

| **Domain** | **Definition^a^** | | **Context** |
| --- | --- | --- | --- |
| *Review question* | A clear and detailed specification of the research question(s) to be addressed by the review. | | Descriptive question – What are the health-related quality of life outcomes experienced post-transplant by adult solid organ transplant recipients? |
| *Epistemology* | The assumptions on the nature of knowledge that underpin the synthesis method and the extent to which these permit the review team to achieve their purpose. | | Constructivist, with the focus on peoples’ lived experiences of health-related quality of life to generate a comprehensive understanding of the outcomes that are experienced post-transplant. |
| *Time/timeframe* | Logistic constraints regarding the expected completion date of the synthesis and the cumulative amount of effort required to deliver the review. | | Nine months; not rapid, but mindful of the need for timely completion of the synthesis to inform next stages in the development of a patient-reported outcome measure. |
| *Resources* | Financial and physical support and infrastructure required to deliver the review. | | Externally funded project with a team of at least three reviewers, with access to support from information specialists. |
| *Expertise* | Knowledge and skill domains required by the review team and the wider network supporting the review. | | Each member of review team highly skilled in evidence synthesis and/or qualitative research. Access to an information specialist for development of the search strategy. |
| *Audience and purpose* | Requirements and expectations of the intended recipients of the review and how review findings are intended to be used. | | Target audiences are academics and health professionals across the health disciplines (e.g. doctors, nurses, allied health professionals). The purpose of the findings is to descriptively highlight and categorise the health-related quality of life outcomes experienced by solid organ transplant recipients. These findings will help health professionals understand how health-related quality of life is impacted post-transplant. They will also inform the development of a patient-reported outcome measure of health-related quality of life, which could be used to identify potential support needs. |
| *Type of data* | The richness, thickness, type (quantitative/qualitative), quality, and quantity of data available to address the review question. | | Original qualitative studies that use recognised qualitative methods of data collection. Large number of studies with heterogeneity in the dimensions of quality of life being explored. |
| *Choice of method* | **Best fit framework synthesis** | **Justification:** This method draws on the advantages of both framework and thematic synthesis. An existing ‘framework’ is deductively populated but without the need to force data that do not ‘fit’ into unsuitable health-related quality of life dimensions. Remaining data are synthesised inductively to develop and incorporate additional dimensions of health-related quality of life until all data are accounted for in a ‘best fit’ framework. Further, including an inductive stage allows us to identify the specific health-related quality of life outcomes that deductively map to the dimensions within the framework. This method is suitable for large numbers of included papers and engages with the literature at a ‘body of evidence’ level that facilitates the intended purpose of this review. Overall, this method is an appropriate approach to answering the review question, and is achievable within the timeframe, with the resources and expertise available in the review team. | |
| ^a^Definitions for each domain taken from *Table 2* within Booth, A., Noyes, J., Flemming, K., et al. (2018). Structured methodology review identified seven (RETREAT) criteria for selecting qualitative evidence synthesis approaches. Journal of Clinical Epidemiology, 99, 41–52. https://doi.org/10.1016/J.JCLINEPI.2018.03.003 | | | |

## Supplementary table 5. Health-related quality of life dimension and outcome definitions

| **Dimension/Outcome** | **Definition** |
| --- | --- |
| **Physical functioning** | **An individual’s physical health and ability to perform functional tasks.** |
| Physical (in)activity/fitness | An individual’s perceived ability to engage in physical activity and exercise. This can be compounded by their perceived fitness levels. |
| Physical capability | An individual’s perception of their general physical functioning (e.g. mobility, flexibility) in day-to-day life. |
| Physical strength | An individual’s general perception of their strength levels and ability to perform functional tasks (e.g. work requirements). |
| **Cognitive functioning** | **An individual’s ability to process thoughts, acquire information, and solve problems (e.g. memory, attention).** |
| Concentration | An individual’s ability to focus and think carefully about what they are doing. |
| Memory impairment | An individual’s deterioration in their ability to store and remember information. |
| **Emotional functioning** | **An individual’s experience, expression, and regulation of positive and negative emotions (e.g. anxiety, frustration).** |
| Altered sense of body | An individual’s perception of, and feeling towards, their body, following receipt of a new organ. This includes attitudes towards incorporating the organ into the body (e.g. organ repair, feelings of alienation, living with a foreign body) and adopting attributes from the donor (e.g. physical, emotional, social attributes). |
| Appreciation of life | An individual’s feelings of joy and value attributed to being alive (e.g. enjoying every day, appreciating the small moments). |
| Body image | An individual’s perception of, and feelings towards, their physical appearance (e.g. hair, weight, scars). |
| Consideration of the donor (family) | An individual’s feelings towards their (known) donor and/or the donor’s family (e.g. processing how the donor family might feel, interest in what the donor was like). |
| Distress | An individual’s experience of general or overall feelings of anxiety or stress. |
| Emotional preparedness | An individual’s perception of whether they were emotionally prepared for their post-transplant lived experiences (e.g. unrealistic expectations, disillusion). |
| Facing death/trauma | An individual’s perception that they were close to death and confronting their mortality, before transplantation provided a ‘resurrection’/‘rebirth’. |
| Fear of cancer | An individual’s concerns regarding their increased risk of cancer due to their levels of immunosuppression. |
| Fear of disease recurrence | An individual’s concerns regarding the potential recurrence of the underlying disease that first led to the transplant. |
| Fear of dialysis | An individual’s feelings towards the potential need to return to dialysis, should they experience graft rejection/failure. This is largely specific to kidney transplant recipients. |
| Fear of graft rejection/failure | An individual’s concerns regarding the potential loss of the graft and the health implications of this. This fear can be exacerbated when waiting for results of graft function tests. |
| Fear of infections | An individual’s concerns regarding their increased risk of infection due to their levels of immunosuppression. |
| Fear of medication side effects | An individual’s concerns regarding the potential side effects of their medication. |
| Fear of missed medication | An individual’s concerns regarding the health implications of non-adherence to their medication. |
| Feeling vulnerable | An individual’s general perception of vulnerability, both overall and in day-to-day life (e.g. medications are a constant reminder of their health situation). |
| Feelings of control/ helplessness | An individual’s perception of how much they feel able to control their lives and health situation. Feelings of helplessness can be compounded by the unpredictable nature of graft rejection. |
| Feelings of isolation | An individual’s feeling of loneliness and isolation (e.g. healthcare, social) following discharge. This can be compounded by reduced/restricted social opportunities and (a lack of) understanding from others regarding the outcomes of transplantation. |
| Feelings of peace | An individual’s general feelings of relaxation and inner peace (e.g. related to mortality, freedom from their antecedent condition). |
| Future planning/changing priorities | An individual’s feelings towards planning for the future (e.g. abandoning long-term ambitions) and a shift in what they consider to be a priority (e.g. favouring time with family over career goals). |
| Future uncertainty | An individual’s concerns about the uncertainty of their future. This is predominantly expressed as an understanding that they/their grafts have a limited, uncertain, life expectancy. |
| Gratitude | An individual’s feeling of gratefulness (e.g. towards the donor, donor family, healthcare professionals, family) for the transplant and all support received. This can be expressed as a desire to give something back (e.g. through charity/community work, by looking after the organ). |
| Guilt | An individual’s feeling of blame, including, for example, that somebody else had to die so that they could live, feeling that they appear ungrateful, that they received their transplant before another transplant candidate. |
| Health perceptions | An individual’s perception of their physical condition (e.g. alleviation of pre-transplant symptoms, feelings of vitality, illness perceptions). |
| Identity/worth | An individual’s sense of self and their perceived value in, and contributions to society (e.g. through occupational and social roles). |
| Low mood | An individual’s expression of a negative emotional state. In severe cases, this can be expressed as depression and/or suicidal ideation. |
| Mood swings | An individual’s experience of rapid fluctuations between positive and negative mood states. |
| New possibilities | An individual’s perception of the physical, social, and role activities they now have the opportunity to engage with, that they could not prior to the transplant. |
| Optimism/hope | An individual’s feelings of hopefulness that they will live long and/or fruitful lives with the transplanted organ. |
| Outlook on life | An individual’s general reflection and evaluation of what is important in life. This includes the perceived importance of being, and determination to be, healthy and positive. |
| Patient burden | An individual’s perceived burden of being a transplant recipient (e.g. due to strict medication regimens, intensive follow-up care, dietary restrictions). |
| Perceptions of transplantation | An individual’s feelings towards being the recipient of a transplant (e.g. regret, relief – especially for those previously on dialysis). |
| Personality/mood changes | An individual’s expression of changes in personality and/or mood following transplantation (e.g. frustration, tolerance levels). |
| Religious/spiritual beliefs | An individual’s spiritual and/or religious orientation and beliefs following transplantation (e.g. increased faith in/stronger relationship with God). |
| Second chance | An individual’s perception that they had been given another opportunity to live their life. This can come with a perceived moral obligation to do everything they can to look after the transplanted organ. |
| Self-confidence/insecurities | An individual’s general insecurities and specific beliefs that they are capable of looking after their new organ, and can overcome any side effects. This can be compounded by the uncertain longevity of the transplant. |
| **Social functioning** | **An individual’s interaction with their social environment (e.g. relationships with partners, family, and friends).** |
| Finding a new partner | An individual’s concerns regarding developing new romantic relationships due to the challenges and limitations imposed by transplantation. |
| Interactions with other transplant recipients | An individual’s interaction with and feelings towards other transplant recipients, including the importance of shared understanding for advice and support. |
| (Lack of) understanding from others | An individual’s perception of other peoples’ understanding of transplantation and related challenges and limitations. A lack of perceived understanding can have emotional, social and role implications. This can be compounded by a feeling that they are being stigmatised. |
| Medication interference | An individual’s experience of the ways that strict medication regimens interfere with day-to-day life and social activities (e.g. day trips). |
| Relationship with partner | An individual’s interaction with their romantic partner/spouse, including support received, how partners were impacted, and relationship dynamics (e.g. housework). |
| Relationships with employer/colleagues | An individual’s interaction with their employer and/or colleagues, including how they are treated and perceptions of available support. |
| Relationships with family | An individual’s interaction with family (e.g. parents, siblings, children), including support received, how family were impacted, and family dynamics (e.g. parenting). |
| Relationships with friends | An individual’s interaction with friends, including support received, maintaining connections, changes in closeness. |
| Risk of infection | An individual’s perception of how their susceptibility to infection influences their engagement in daily activities (e.g. crowded spaces). |
| Risk of skin cancer | An individual’s perception of how their susceptibility to skin cancer influences their engagement in social activities (e.g. travel in Summer). |
| Social opportunities/ restrictions | An individual’s perception of the social activities that are possible once transplant-related challenges and limitations (e.g. medication, risk of infection, dietary restrictions) have been considered. |
| **Role functioning** | **An individual’s ability to perform their occupational and social roles (e.g. work, housework, leisure activities).** |
| Ability to work | An individual’s perceived ability to perform their occupational role, including the required functional tasks. |
| Autonomy | An individual’s feelings of freedom to make their own decisions (e.g. no longer restricted by dialysis machine). |
| Driving | An individual’s perceived ability and confidence to drive a vehicle. |
| Financial implications | An individual’s concerns related to the costs of the transplant and its treatment, and the implications of changes in employment. |
| Finding employment/ employability | An individual’s concerns about their job prospects and experience of job seeking, due to their health situation and the demands of follow-up care. |
| Hobbies/leisure activities | An individual’s perceived ability to pursue and engage in new/existing hobbies and leisure activities (e.g. gardening, playing music). |
| Household tasks | An individual’s ability to perform domestic tasks (e.g. cooking, cleaning). |
| (In)dependence | An individual’s perceived ability to live (aspects of) their life without help or influence from other people. |
| Returning to ‘normal’ | An individual’s general perception of their ability to return to ‘normal’ (i.e. resume/regain occupational and social roles). |
| Returning to work/changes in employment | An individual’s experiences of, and perceived ability to return to work, and consequent changes in employment, where returning to work was not possible. |
| Self-care | An individual’s ability to engage in self-care (e.g. personal hygiene, eating, drinking). |
| Travelling | An individual’s perceived ability and attitudes towards taking trips/going on holiday (abroad). |
| **Sexual functioning** | **An individual’s ability to react sexually or to experience pleasure sexually.** |
| Attitudes towards sexual activity | An individual’s change in their perception of, and value given to sexual activity, and its importance in returning to ‘normal’. |
| Interest in sexual activity | An individual’s desire and libido to be intimate and participate in sexual activities. |
| Reproduction/fertility | An individual’s ability to think about having children and starting a family as a transplant recipient. |
| Sexual capability | An individual’s physical ability to be sexually active and experience pleasure sexually (e.g. orgasm, erections, vaginal lubrication). |
| Sexual fears/concerns | An individual’s doubt in their sexual ability and concerns that sexual activity would have physical consequences for their transplant. |
| **Symptom distress** | **An individual’s experience of physical or emotional discomfort due to the presence of a symptom (e.g. pain).** |
| Breathlessness | An individual’s experience of shortness of breath. |
| Comorbidities | An individual’s experience of additional health conditions alongside the transplant (e.g. heart disease). |
| Changes in appetite | An individual’s experience of appetite loss or hunger pangs, and general control over their appetite. |
| Gastrointestinal symptoms | An individual’s experience of gastrointestinal-related symptoms (e.g. nausea, constipation, diarrhoea, vomiting). |
| Hair growth/loss | An individual’s experience of hair loss and/or unwanted hair growth on other body parts (e.g. limbs, face). |
| Hypertension | An individual’s experience of high blood pressure. |
| Infections | An individual’s experience of developing infections. |
| Musculoskeletal symptoms | An individual’s experience of musculoskeletal-related symptoms (e.g. muscular spasms). |
| Neurological symptoms | An individual’s experience of neurological-related symptoms (e.g. tremors, headaches, neuropathy). |
| New onset diabetes | An individual’s experience of developing new onset diabetes and their feelings towards the implications of being diabetic. |
| Oral symptoms | An individual’s experience of oral-related symptoms (e.g. swollen gums, swallowing). |
| Osteoporosis | An individual’s experience of weakened bones. |
| Pain/discomfort | An individual’s general experience of pain and discomfort (e.g. aches, stabbing sensations). |
| Scars | An individual’s concerns related to the scarring from their transplant operation. |
| Skin symptoms | An individual’s experience of skin-related symptoms (e.g. acne, fragile skin). |
| Swelling | An individual’s experience of body swelling (e.g. legs and feet). |
| Urogenital symptoms | An individual’s experience of urogenital-related symptoms (e.g. uterine growths). |
| Visual impairment | An individual’s experience of deterioration in their eyesight. |
| Weight gain/loss | An individual’s experience of , and feelings towards changes in weight (gain and loss), including the influence on their appearance and physical function. |
| **Fatigue** | **An individual’s overall feeling of tiredness or lack of energy.** |
| Chronic fatigue | An individual’s experience of persistent and ongoing feelings of fatigue. |
| Energy/stamina | An individual’s perceived (ability to sustain) levels of energy to engage in daily life. |
| Tiredness | An individual’s feelings of lethargy and exhaustion, and need for rest. |
| **Sleep** | **An individual’s ability to get enough sleep or good sleep quality.** |
| Hallucinations/dreams | An individual’s experience of vivid dreams and false perceptions of reality. |
| Sleep difficulties | An individual’s challenges with getting to sleep or getting enough sleep (e.g. due to insomnia). |
| Sleep disturbances | An individual’s experience of sleep disruptions (e.g. medication timing, muscular spasms). |
| ^a^Definitions for each health-related quality of life outcome were determined by the review authors to appropriately reflect the data within the findings. | |

## Supplementary table 6. Summary of sample characteristics across all included studies

| **Characteristic^a,b,c^** | **Combined sample size** | **Included studies^j^** |
| --- | --- | --- |
| **Overall** | **n = 689** | **n=39** ^[37–70,72–76]^ |
| *Organ received^d^* | *n = 700* | *n=39* ^[37–70,72–76]^ |
| Kidney | n = 334 | n=18 ^[37–50,73–76]^ |
| Liver | n = 119 | n=13 ^[51–60,74–76]^ |
| Heart | n = 156 | n=12 ^[61–68,73–76]^ |
| Lung | n = 86 | n=6 ^[69,70,72–75]^ |
| Pancreas | n = 5 | n=1 ^[73]^ |
| *Sex* | *n = 664* | *n=37* ^[37–47,49–56,58–70,72–76]^ |
| Women | n = 272 | n=37 ^[37–47,49–56,58–70,72–76]^ |
| Men | n = 392 | n=37 ^[37–47,49–56,58–70,72–76]^ |
| *Ethnicity^e^* | *n = 114* | *n=7* ^[40,44,45,47,52,54,60]^ |
| White | n = 86 | n=7 ^[40,44,45,47,52,54,60]^ |
| Other | n = 28 | n=4 ^[40,47,52,60]^ |
| *Relationship status^f^* | *n = 328* | *n=20* ^[37–39,41,44,46,47,50,52,54,55,59,64,66–70,72,76]^ |
| Married | n = 234 | n=20 ^[37–39,41,44,46,47,50,52,54,55,59,64,66–70,72,76]^ |
| Single | n = 67 | n=16 ^[37,39,41,44,46,47,50,52,55,59,66–68,70,72,76]^ |
| Divorced | n = 19 | n=6 ^[37,41,46,47,55,70]^ |
| Widowed | n = 8 | n=4 ^[47,54,55,70]^ |
| *Education^g^* | *n = 301* | *n=13* ^[38,39,41,43,47,50,59,64,66,68–70,76]^ |
| Illiterate | n = 7 | n=4 ^[39,50,59,76]^ |
| Primary education | n = 96 | n=11 ^[38,39,41,43,47,50,59,64,66,69,70]^ |
| Secondary education | n = 70 | n=9 ^[38,39,43,47,50,64,66,68,70]^ |
| Further or higher education | n = 92 | n=10 ^[38,39,41,43,47,59,66,69,70,76]^ |
| University (Bachelors) | n = 30 | n=8 ^[41,43,47,59,66,68,69,76]^ |
| University (Masters) | n = 6 | n=2 ^[41,76]^ |
| *Employment status^h^* | *n = 242* | *n=18* ^[39,41,43,44,46,47,51,52,54,55,59,64,66,68,69,72,74,76]^ |
| Employed | n = 99 | n=16 ^[39,41,43,44,46,47,51,52,54,55,64,66,68,69,74,76]^ |
| Self-employed | n = 6 | n=2 ^[41,59]^ |
| Unemployed | n = 30 | n=6 ^[39,41,44,54,64,68]^ |
| Retired | n = 62 | n=10 ^[44,46,47,51,52,54,59,66,72,74]^ |
| Unable to work | n = 16 | n=4 ^[46,51,52,55]^ |
| Housewife | n = 24 | n=5 ^[41,59,66,68,76]^ |
| Student | n = 5 | n=3 ^[41,55,74]^ |
| *Rurality^i^* | *n = 101* | *n=6* ^[38,39,47,59,60,76]^ |
| Urban | n = 86 | n=6 ^[38,39,47,59,60,76]^ |
| Rural | n = 15 | n=3 ^[39,47,59]^ |
| ^a^Note that the terminology used to label the characteristics is informed by what was reported within the included studies.  ^b^Due to the heterogeneity in how age and time since transplant were reported (e.g. mean, median, range, groups), we were unable to accurately summarise these characteristics.  ^c^Due to the limited number of studies (n=4) reporting co-morbid conditions, and the heterogenous range of conditions reported, it was not appropriate to summarise these here.  ^d^Where participants received more than one organ (n=9) (e.g. simultaneous pancreas-kidney) they have been counted once for each organ.  ^e^‘White’ includes White, White British, Caucasian, and New Zealand European. ‘Other’ includes Caribbean, Egyptian, Hispanic, Indian, Maltese, Māori, and those reported as ‘Other’ within studies; each of these ethnicities were not reported in more than one study.  ^f^‘Married’ also includes ‘Stable relationship’, ‘In a relationship’ and ‘Living with a partner/spouse’. ‘Single’ also includes ‘Unmarried’, ‘Not living with a partner/spouse’, and ‘Live alone’. ‘Divorced’ also includes ‘Separated’.  ^g^‘Primary education’ also includes ‘Below matric’ and ‘School level’. ‘Secondary education’ also includes ‘Middle school’, ‘High school’ and ‘Matric and 12^th^’. ‘Further or higher education’ also includes ‘Diploma’, ‘Vocational’ and ‘Graduate’. ‘University (Bachelors)’ also includes ‘University degree’ and ‘Associate degree’.  ^h^‘Employed’ includes full-time and part-time, as studies were not typically explicit. ‘Unable to work’ also includes ‘Ill-health retirement’, ‘Disability payments’, and ‘Disability pension’. ‘Housewife’ was an employment status explicitly reported by study authors. The possibility cannot be discounted that ‘Employed’ and ‘Retired’ may contain those ‘Self-employed’ or ‘Unable to work’, respectively, but authors were not explicit.  ^i^‘Urban’ also includes ‘Metropolitan’ and ‘Semi-urban’. ‘Rural’ also includes ‘Nonmetropolitan’.  ^j^The citation numbers listed here can be cross-referenced to the reference list in the main text. | | |

## Supplementary table 7. Additional sample characteristics

| **Study** | **Education** | **Employment status^a^** | **Rurality** | **Co-morbid conditions** |
| --- | --- | --- | --- | --- |
| *Kidney recipients* | | | | |
| Abarca-Durán (2021) | NR | NR | NR | NR |
| de Brito (2015) | Primary education (n=26), Secondary education (n=10), Higher education (n=14) | NR | Urban (n=13), NR (n=37) | NR |
| Dutta (2023) | Illiterate (n=1), Below matric (n=6), Matric and 12th (n=3), Graduate (n=8) | Employed (n=12), Unemployed (n=6) | Urban (n=7), Rural (n=10), Semi-urban (n=1) | Hypertension (n=9), Diabetes (n=5), Chronic liver disease (n=2), Heart disease (n=1) |
| Jones (2020) | NR | NR | NR | NR |
| Kamran (2016) | School level (n=4), Graduation (n=2), Bachelors (n=3), Masters (n=5) | Employed (n=4), Housewife (n=4), Self-employed (n=2), Student (n=2), Unemployed (n=2) | NR | NR |
| Kristensen (2021) | NR | NR | NR | NR |
| Luk (2004) | Primary education (n=15), Secondary education (n=9), Higher education (n=2), Bachelors (n=5) | Employed (n=20: Full-time n=12, Part-time n=8), NR (n=11) | NR | NR |
| Ó Lonargáin (2017) | NR | Employed (n=3), Retired (n=2), Unemployed (n=1) | NR | NR |
| Orr (2007) | NR | NR | NR | NR |
| Petre (2021) | NR | Ill-health retirement (n=6), Employed (n=1), Retired (n=1) | NR | NR |
| Pinter (2017) | Primary education (n=6), Secondary education (n=8), Higher education (n=6), Diploma (n=7), University degree (n=3) | Retired (n=26), Part-time (n=4) | Metropolitan (n=27), Nonmetropolitan (n=3) | Diabetes (n=16), Cardiovascular disease (n=12), Cancer (n=12), Infection (n=10), Rheumatologic disorder (n=9), Neurologic disorder (n=9), Gastrointestinal conditions (n=6), Ophthalmologic condition (n=4), Bone disease (n=3), Respiratory disease (n=3), Urologic condition (n=2) |
| Santos (2016) | NR | NR | NR | NR |
| Schipper (2014) | NR | NR | NR | NR |
| Tasdemir (2023) | Illiterate (n=3), Primary education (n=8), Secondary education (n=6) | NR | NR | NR |
| *Liver recipients* | | | | |
| Bardet (2014) | NR | Employed (n=3), Retired (n=4), Unable to work (n=1) | NR | NR |
| Bean (2005) | NR | Disability payments (n=5), Employed (n=3), Retired (n=4) | NR | NR |
| Beckmann (2022) | NR | NR | NR | Weight: Obesity (n=4), Overweight (n=4) |
| Dudley (2007) | NR | Employed (n=3), Unemployed (n=3), Retired (n=2) | NR | Renal dysfunction (n=4), Hypertension (n=2), Diabetes (n=2), Arthritis, Chronic bronchitis, Chronic obstructive airways disease, Recurrent pleural effusion, Hypogammaglobulinemia, Epilepsy (n=1 each) |
| Forsberg (2000) | NR | Employed (n=7), Student (n=1), Disability pension (n=4) | NR | NR |
| Naden (2023) | NR | NR | NR | NR |
| Robertson (1999) | NR | NR | NR | NR |
| Sargent (2007) | NR | NR | NR | NR |
| Taher (2021) | Illiterate (n=2), Primary education (n=6), Diploma (n=7), Bachelors (n=3) | Self-employed (n=4), Housewife (n=7), Retired (n=2), Other (n=5) | Urban (n=16), Rural (n=2) | NR |
| Wainwright (2018) | NR | NR | Urban (n=7), Unclear (n=10) | NR |
| *Heart recipients* | | | | |
| Almgren (2017) | NR | NR | NR | NR |
| Bahramnezhad (2022) | NR | NR | NR | NR |
| Dehghan-Nayeri (2017) | NR | NR | NR | NR |
| Fatma (2021) | Primary education (n=5), Middle school (n=3), High school (n=3) | Employed (n=1), Unemployed (n=10) | NR | NR |
| Kaba (2005) | NR | NR | NR | NR |
| Pashaeypoor (2021) | Primary education (n=2), Middle school (n=2), Diploma (n=6), Bachelor (n=3) | Employed (n=4), Housewife (n=5), Retired (n=4) | NR | NR |
| Peyrovi (2014) | NR | NR | NR | NR |
| Yoo (2021) | Secondary education (n=5), University (n=10) | Employed (n=6), Unemployed (n=8), Housewife (n=1) | NR | NR |
| *Lung recipients* | | | | |
| Graarup (2017)^b^ | Primary education (n=11), Vocational (n=6), Short-cycle higher education (n=8), Medium-cycle higher education (n=2), University degree (n=2) | Employed (n=9), NR (n=22) | NR | NR |
| Seiler (2016); Eichenlaub (2021)^c^ | Primary education (n=7), Secondary education (n=21), Higher education (n=12) | NR | NR | NR |
| Thomsen (2009) | NR | Retired (n=10) | NR | NR |
| *Mixed samples of organ recipients* | | | | |
| Boaz (2014) | NR | NR | NR | NR |
| Forsberg (2016) | NR | Employed (n=11), Retired (n=7), Student (n=2) | NR | NR |
| Nilsson (2008) | NR | NR | NR | NR |
| Sheikhalipour (2018) | Illiterate (n=1), Diploma (n=12), Associate degree (n=1), Master degree (n=1) | Employed (n=8), Housewife (n=7) | Urban (n=15) | NR |
| *NR = Not reported*  ^a^‘Housewife’ was an employment status explicitly reported by all studies where this is stated.  ^b^Graarup (2017) reported sample characteristics for n=31 lung recipients, but only interviewed n=26, so education and employment status may not be accurate.  ^c^Eichenlaub (2021) is a secondary analysis of the data collected for Seiler (2016), but did not include two of the participants. Where available, the sample characteristics reported here are for the full dataset (n=40). | | | | |

## Supplementary table 8. Author-reported key findings

| **Study** | **Author-reported key findings** |
| --- | --- |
| *Kidney recipients* | |
| Abarca-Durán (2021) | **Themes:** The impact of a kidney transplant on sexuality (Sexuality is relegated to the background; Physical decline acts as a sexual inhibitor; Changes in sexual activity following a kidney transplant); Sexual education in kidney transplant recipients (Sexuality: a hidden concern amongst kidney transplant recipients; Talking about sexuality with healthcare professionals). |
| de Brito (2015) | **Principal positive changes:** Return to activities; Freedom/independence; Well-being and health; Strengthening of the I; Closening of interpersonal relationships. **Most-cited stressors:** Fear; Medication; Excess of care/control; Specific characteristics of the treatment; Failure to return to the social roles. |
| Dutta (2023) | **Themes:** Relief from dialysis‑associated problems; Perceived social neglect before and after transplant; Burden of posttransplant regimen; Impact on occupation; Attitude toward donor; Posttransplant lifestyle modifications; Concerns about future quality of life; Effect on family and children; Facilitating agents in transplant process. |
| Jones (2020) | **Themes:** I am living with a 'foreign body' inside me (Fear/feelings about the kidney itself; Survivors' guilt; Feelings of isolation; Impacts of medication); Why am I distressed? (Expectations of living with a transplant; Coping with distress; Lack of information about transplants and support); Different patients want different types of support. |
| Kamran (2016) | **Themes:** Optimism; Gratitude; Self-identity; Emotional morbidity; Transplant-related fears and anxieties and future concerns. |
| Kristensen (2021) | **Themes:** Experiencing body vulnerability while getting back to life; Feeling guilt while experiencing gratitude; Living in limbo while one's identity is changing; Facing the future with hope while having reservations. |
| Luk (2004) | **Categories:** Physical-psychological health status (Physical capability; Impact of side effects of medication on lifestyle; Compliance); Social life (Relationship with others; Recreation); Financial status (Employability; Medical costs); Patient needs (Expectations about advice and information; Source of information). |
| Ó Lonargáin (2017) | **Themes:** Discovering a new life; Experiencing support from others; Experiencing challenges post-transplant; Remembering the donor and the donor's family. |
| Orr (2007) | **Themes:** Medicalisation; Fear; Gratitude and sense of obligation; Coping. |
| Petre (2021) | **Themes:** An inner struggle to find a new normality (personal); Me and the others (relational); Only between me, my donor and god (spiritual). |
| Pinter (2017) | **Themes:** Restoring vitality of youth (Revived mindset for resilience; Embracing enjoyment in life; Drive for self-actualisation); Persisting through prolonged recovery (Yielding to aging; Accepting functional limitations; Pushing the limit; Enduring treatment responsibilities); Imposing sicknesses (Combatting devastating comorbidities; Painful restrictions; Emerging disillusionment; Anxieties about accumulating side effects; Consuming treatment burden); Prioritizing graft survival (Privileged with a miracle; Negotiating risks for longevity; Enacting a moral duty; Preserving the last opportunity); Confronting health deterioration (Vulnerability and helplessness; Narrowing focus to immediate concerns; Uncertainty of survival); Value of existence (Purpose through autonomy; Refusing the burden futile treatment; Staying alive by all means). |
| Santos (2016) | **Themes:** Similarity to "normal" living: advantages resulting from kidney transplant; Permanence of chronic condition and the need for care after surgery: disadvantages experienced by kidney transplant recipients |
| Schipper (2014) | **Themes:** Experiences before transplantation (Limitations and lack of freedom; Various emotions: hope, anger, jealousy, and doubt); Positive improvements going together with adaptation problems (Improved physical condition and regained freedom; Adaption difficulties: daily activities, role functioning, social relations; Gratefulness; Trust or fear; Guilt); Less positive changes going together with adaptation problems (Negative changes and side effects; Expectations and disillusions; Disillusions, gratefulness and guilt; Expectations of environment); Adaptation and resilience after transplantation. |
| Tasdemir (2023) | **Themes:** Problems experienced by kidney transplant recipients (Management of drug therapy; Role-relationship management; Permanent treatment responsibilities and burden); Inevitable fears (Fear of rejection; Fear of returning to dialysis treatment; Pervasive fear of routine examination results); Situations that only individuals with kidney transplants can understand - "Only the sufferer knows" (Significance of kidney transplant surgery; Sense of gratitude towards the donor; Being 'sick' in the eyes of others; Not being understood by others). |
| *Liver recipients* | |
| Bardet (2014) | **Themes:** Transplantation and graft rejection representations (Transplantation identity; Transplantation consequences; Graft rejection identity; Graft rejection causes; Graft rejection consequences; Time line); Immunosuppressant medication beliefs (Necessity; General beliefs; Complexity of medication regimen; Medication cost); Social dimension (The patient-family relationship; The patient-doctor relationship; Patient information source; The emotional response). |
| Bean (2005) | **Positive aspects:** Ability to work again; Broadened spiritual perspective; Desire to give back to society; Enhanced appreciation and value of family; New perspective of enjoying life to the fullest; Peace about end of life; Restored or improved health. **Negative aspects:** Cognitive impairments (memory, thinking); Fatigue; Fear of future impairments; Fear of organ rejection; Financial stressors; Inability to work; Medication side effects |
| Beckmann (2022) | **Themes:** The main thing is to be alive; The arduous path back to living (Surviving a suffering period of the illness; The joy of being given the gift of a second life); A pleasurable new phase of life (Experiencing a sudden, unexplainable weight gain; To finally look healthy again); I am allowed to look like this now (Body weight becomes a source of stress; Losing weight requires the right headspace; All attempts to lose weight fail). |
| Dudley (2007) | **Themes:** Longer term health problems (The early post-transplant period; Disappointment; the 12 month turning point; Adapting to longer-term limitations; Modest expectations); The stigma of liver disease (Alcohol; Embarrassment; Guilt); Living with uncertainty (Pragmatism; Recurrent disease; Organ rejection; Optimism); A new outlook on life (Seeing each day as a bonus; Taking care of their health; A life-changing experience; A second chance); The donor and donor's family (Gratitude; Contact with the donor's family; Guilt; Indebtedness). |
| Forsberg (2000) | **Categories:** Facing the inevitable (Acceptance; Joy and anticipation; Grief; Security; Feeling guilty; Unprepared and scared); Recapturing the body (Master the body; Fighting fatigue; Overcome bodily weakness; Being helpless and dependent; Losing appetite; Rediscover sexuality); Emotional chaos (Contradictory feelings; Becoming a new person; Guilt and gratitude; Anxiety and fear); Leaving the experts (Feeling abandoned; Feeling like a stranger; Losing dependency; Doubting medical knowledge; Lack of self-care capacity; Enjoying going home); Family and friends (Family involvement; Marital support; Being a heroine; Meeting the demands; Dealing with solitude); The threat of graft rejection (No big deal; Fear of death; Constant awareness of the body; Constant fear; An invisible threat; The betrayal of the body); Honour the donor (Being indebted to someone unknown; Treasure the gift; Protecting the gift; Being grateful). |
| Naden (2023) | **Themes:** From great suffering to gratitude and a humble attitude toward life; From living in uncertainty to leading a normal life; From hopelessness and anxiety of pain to an indifferent attitude towards life. |
| Robertson (1999) | **Categories:** Pre-transplant themes; Post-transplant themes; Personality; Incentives; Liver transplant unit support - staff and other patients. |
| Sargent (2007) | **Themes:** Inactivity (Fatigue; Lethargy; Immobility; Weight loss; Feeling helpless); Health restoration (Physical recovery; Regaining autonomy; Adapting to immunosuppression); Modification (Lifestyle changes; Role models); Introspection (Acceptance; Social isolation; Reciprocation; Fear of infection). |
| Taher (2021) | **Categories:** Self-care deficit (Limited self-care knowledge; Gradual decline of self-care); The need for seeking information (Lack of a reliable source of information; Seeking strategies to change care-related approach); Fears and concerns (Concerns over inability to afford care; Fear of death); Hope-despair duality (Hope; Despair). |
| Wainwright (2018) | **Themes:** Shifting priorities; Reclaiming independence and normalcy. |
| *Heart recipients* | |
| Almgren (2017) | **Themes:** Doubting survival (Brooding about the survival of the graft; Not knowing how long one will live; Preparing for the worst); Doubting the recovery process (Questioning one's current health condition; Fearing illness or disease; Coping with setbacks and test results); Doubting one's performance (Struggling and moving on; Coping with restrictions or prescriptions; Avoiding looking back); Struggling with close relationships (Playing a role; Being disappointed in family and friends; Experiencing partner's disappointment); Feeling abandoned (Not taken seriously; Lacking knowledge and experience; Lacking support from healthcare professionals); Doubting the future (Worrying about one's financial situation; Worrying about returning to work). |
| Bahramnezhad (2022) | **Categories:** Religious concerns (Religious commitment; Guilt); Sense of duality (Fear of the unknown and the known; Liberation or captivity); Emotional chaos (Seeking identity; Self-alienation; Self-conflict); Additional concerns (Physical problems and social problems; Financial problems; Social problems); Toleration and management (Fatalism; Transformation of understanding of life; Conscious forgetfulness). |
| Dehghan-Nayeri (2017) | **Main theme:** Living under the perfect storm of problems. **Categories:** Financial distress (The shadow of financial strain on life; The inefficiency of financial support systems; Struggle for managing the expenses); Perceived physical and psychosocial changes (Physical crisis; Inability to perform roles; Psychological problems); Greater commitment to one's own health (Accepting responsibility for health; Information seeking); Tendency to spirituality (Reinforcement of religious beliefs and values; Gratefulness to the donors). |
| Fatma (2021) | **Themes:** A new life with a new heart (Opportunity arising at an unexpected time; Heart donation and transplantation as a value); Challenges (Management of medication therapy and side effects; Emotional management; Maintaining social relations; Social stigma); Coping (Faith in God and praying; Responsibility of living with a blessing (donated heart); Health professional support; Family support; Peer counselling). |
| Kaba (2005)^a^ | **Categories:** Traumatic experience; That's a small price to pay for being alive; Somebody else's heart inside me (I'm very grateful to the person who gave me the heart; What happened to my old heart?; Will the new heart change me?); Coping. |
| Pashaeypoor (2021) | **Transition stage:** Emotional turmoil (Feeling guilty; Alienation with the new heart; Gratitude); Feeling of duality (Personality change; Lifestyle change). **Stabilisation stage:** Self-acceptance (Accepting the new heart; Feeling of unity with the donor; Normalisation). |
| Peyrovi (2014) | **Themes:** A new life (New vision about life; Pleasure from every moment of life); Living with vigilance (You must be very careful; You must be under follow-up; Being under supervision of family); Living with new concerns (Impact on life affairs; Being trouble for family; Difficulty with follow-up treatment; Complications of transplant; Being under pressure to take medications; Relationship with donor, donor's family, and other heart transplant recipients); Bearing others’ behaviours (Stigmatisation; Frequent recommendations; Frequent questions; Myths; Irony of hearing); Paradoxical emotions (Desire to be like others vs being unique; Hope vs despair; Drugs: good vs bad; Fear vs carelessness; Combined feelings of concern and abandon; Regret vs satisfaction; Death wish vs desire to be alive); Prominent role of God in life (Intimacy with God; Entrusting self to God; Accepting the faith; Deeper belief in God; Trying to perform religious obligations). |
| Yoo (2021) | **Themes:** Unwelcome rebirth without vitality (Lower expectations from new life; Incompatible with physical and emotional changes from drug side effects); Facing unreachable ordinary tasks in life (Feeling of alienation in social relationships; High barrier to job switch; Ambivalence to marriage and childbirth); Lifestyle bordering between burdensome and self-valued (Focusing on negative aspects; Focusing on my own life); Finding the true meaning of a newly given life (Recognising the value of the family; Being satisfied with the life given; Taking a step further into a worthy life). |
| *Lung recipients* | |
| Graarup (2017) | **Themes:** Receiving a lung transplant is an overwhelming experience (Gaining physical capacity; Energy to be part of family; Struggling to recover); Being troubled by physical and psychological challenges (Medical side effects; Postoperative complications; Psychological problems); A balance between joy and challenges (New chance in life; Worry about the future). |
| Seiler (2016); Eichenlaub (2021) | **Seiler: Clusters:** Thoughts, feelings, and attitudes about the transplantation process itself (Physical benefits; Physical complaints; Emotional distress; Psychological benefits); Thoughts, feelings, and attitudes about the new lungs (Fear of transplant rejection; Thoughts regarding new lungs; Satisfaction with new lungs); Thoughts, feelings, and attitudes about the medication regimen (Side effects; Benefits of medical treatment; Restrictions in everyday life; Adherence to medication).  **Eichenlaub:** Transplantation complex (The donor is killed; The body is broken open; Objects penetrate into the body/are devoured; The donor is the recipient; The donor is a part of the recipient's internal world; The (new) object is a member of the family of organs; The new organ (lung) can be expelled); Axis of psychosomatic totality (Symbolic pole; Asymbolic pole; Organic pole; Imaginary zone). |
| Thomsen (2009) | **Themes:** A second chance; An ordinary life without chronic rejection; Even minor daily activities take time with chronic rejection; Need for support and knowledge. |
| *Mixed samples of organ recipients* | |
| Boaz (2014) | **Key concerns:** Challenges in regaining normality, both at three months for those experiencing acute and distressing side effects, and later relating to the long-term side effects of transplant medication and co-morbidities. Most dramatic threat to normality (disrupted normality) came from episodes of rejection and transplant failure. |
| Forsberg (2016) | **Categories:** Deconstruction (Accepting the severe illness; Slowing down the pace; Stop working; Doing nothing; Putting life on hold; Isolation; Forcing oneself to take part in activities; Appreciating the support from friends); Restriction (Adhering to restrictions; Preparing for hospitalisation; Enduring adversities; Enjoying vitality; Mourning losses; Approaching one's employer); Reconstruction of social function (Choosing your own company; Meeting friends; Working full time; Appreciating life differently; Enjoying travelling; Being sexually intimate; Acknowledging the burden of the caregiver - the spouse/co-habitant). |
| Nilsson (2008) | **Domains:** Abstract threat to life (Making something concrete); Concrete threat to health (Emotional; Somatisation; Medical); Trust in the body (Awareness of the body; Body related; Bodily failure); Striving to control the threat (Failure; Fatalistic; Compliance; Fitness; Avoidance; Denial; Lucky at games); One's identity (Empowerment; Comfortable; Normification; Identity adjustment; Normalisation). |
| Sheikhalipour (2018) | **Themes:** Back from the grave (Organ as the God's deposit; God as the source of life; New life); Chapter of prosperity (Spring of the body; Recovery; Peace and joy; Benevolent and good behaviour; Renewal; Opportunity of being together again; Golden age; Positive perspective; The sense of normality; The return of health; Spiritual evolution); The fall (A lack of energy; The mirage of transplantation; Hell on the earth). |
| This table presents the themes (subthemes), categories (subcategories), clusters, domains, and key concerns, as reported by authors of included studies, prior to interpretation in the data synthesis.  ^a^Kaba (2005) report four categories but only present findings for ‘Somebody else’s heart inside me’. | |

## Supplementary table 9. Qualitative findings mapped to health-related quality of life dimensions

| **Study** | **Number of HRQoL outcomes** | **Health-related quality of life dimensions** | | | | | | | | | |
| --- | --- | --- | --- | --- | --- | --- | --- | --- | --- | --- | --- |
|  |  | *Physical functioning* | *Cognitive functioning* | *Emotional functioning* | *Social functioning* | *Role functioning* | *Sexual functioning* | *Symptom distress* | *Fatigue* | *Sleep* | **Total** |
| *Kidney recipients* | | | | | | | | | | | |
| Abarca-Durán (2021) | 13 |  |  | + | + |  | + | + |  |  | 4 |
| de Brito (2015) | 20 | + |  | + | + | + |  | + | + |  | 6 |
| Dutta (2023) | 19 | + |  | + | + | + |  |  |  |  | 4 |
| Jones (2020) | 20 |  |  | + | + | + |  | + |  |  | 4 |
| Kamran (2016) | 33 |  |  | + | + | + | + | + | + |  | 6 |
| Kristensen (2021) | 24 |  |  | + | + | + |  | + | + |  | 5 |
| Luk (2004) | 33 | + | + | + | + | + |  | + |  | + | 7 |
| Ó Lonargáin (2017) | 29 | + |  | + | + | + |  | + |  |  | 5 |
| Orr (2007) | 33 |  |  | + | + | + |  | + | + |  | 5 |
| Petre (2021) | 42 |  |  | + | + | + | + | + |  |  | 5 |
| Pinter (2017) | 41 | + |  | + | + | + |  | + | + |  | 6 |
| Santos (2016) | 24 | + |  | + | + | + |  | + |  |  | 5 |
| Schipper (2014) | 27 |  |  | + | + | + | + | + | + |  | 6 |
| Tasdemir (2023) | 14 |  |  | + | + | + |  |  |  |  | 3 |
| *Liver recipients* | | | | | | | | | | | |
| Bardet (2014) | 26 |  |  | + | + | + |  |  | + |  | 4 |
| Bean (2005) | 23 |  | + | + | + | + |  |  | + |  | 5 |
| Beckmann (2022) | 23 | + | + | + | + | + |  | + | + |  | 7 |
| Dudley (2007) | 21 | + |  | + | + | + |  | + | + |  | 6 |
| Forsberg (2000) | 28 |  |  | + | + | + | + | + | + | + | 7 |
| Naden (2023) | 20 | + |  | + |  | + |  |  | + | + | 5 |
| Robertson (1999) | 13 |  | + | + | + | + |  |  | + |  | 5 |
| Sargent (2007) | 27 | + |  | + | + | + |  | + | + |  | 6 |
| Taher (2021) | 16 |  |  | + | + | + | + | + |  |  | 5 |
| Wainwright (2018) | 23 |  |  | + | + | + |  |  |  |  | 3 |
| *Heart recipients* | | | | | | | | | | | |
| Almgren (2017) | 21 |  |  | + | + | + |  |  | + |  | 4 |
| Bahramnezhad (2022) | 16 | + |  | + | + | + | + |  | + |  | 6 |
| Dehghan-Nayeri (2017) | 22 | + |  | + | + | + |  | + |  | + | 6 |
| Fatma (2021) | 19 |  |  | + | + |  |  | + |  |  | 3 |
| Kaba (2005) | 6 |  |  | + |  |  |  |  |  | + | 2 |
| Pashaeypoor (2021) | 9 |  |  | + |  | + |  |  |  |  | 2 |
| Peyrovi (2014) | 22 |  |  | + | + | + | + | + |  |  | 5 |
| Yoo (2021) | 36 |  | + | + | + | + | + | + | + | + | 8 |
| *Lung recipients* | | | | | | | | | | | |
| Graarup (2017) | 35 | + |  | + | + | + |  | + | + | + | 7 |
| Seiler (2016) | 46 | + |  | + | + | + |  | + | + | + | 7 |
| Eichenlaub (2021) | 13 |  |  | + | + |  |  | + |  | + | 4 |
| Thomsen (2009) | 43 | + |  | + | + | + | + | + | + |  | 7 |
| *Mixed samples of organ recipients* | | | | | | | | | | | |
| Boaz (2014) | 42 | + |  | + | + | + | + | + | + | + | 8 |
| Forsberg (2016) | 16 | + |  | + | + | + | + |  | + |  | 6 |
| Nilsson (2008) | 12 | + |  | + | + |  |  |  | + |  | 4 |
| Sheikhalipour (2018) | 20 | + |  | + | + | + |  |  | + |  | 5 |
| **Total** | | 19 | 5 | 40 | 37 | 35 | 12 | 26 | 24 | 10 |  |
| ‘+’ means that the qualitative findings reported by an included paper have been mapped to the health-related quality of life dimension in our best fit framework synthesis. | | | | | | | | | | | |

## Supplementary table 10. Health-related quality of life dimensions and outcomes with supporting quotes

| **HRQoL dimensions** | **HRQoL outcomes** | **Papers with  related findings** | **Example supporting quotes** |
| --- | --- | --- | --- |
| ***Physical functioning*** | | **n=19** |  |
|  | - Physical (in)activity/fitness | n=14 | - “I used to be able to walk a long way…now it seems I’ve only got to walk half way and I’m out of breath…I take the dog for a walk in the morning and I get to the top of the road and I have to stop” – Dudley (2007) |
|  | - Physical capability | n=10 | - “I left hospital in a wheelchair…It took about three months to walk properly without having to take a rest every ﬁve steps.” – Sargent (2007) |
|  | - Physical strength | n=13 | - “I will no longer be able to lift much weight and do the same thing as before transplant. If I want to do any work, I will do light work only.” – Dutta (2023) |
| ***Cognitive functioning*** | | **n=5** |  |
|  | - Concentration | n=2 | - “I want to study or work more, but when I try, I get tired and I cannot concentrate. Do you know what it is like to feel your mind and body sinking below the surface?” – Yoo (2021) |
|  | - Memory impairment | n=3 | - “My memory isn't quite what it used to be but I'm not complaining.” – Robertson (1999) |
| ***Emotional functioning*** | | **n=40** |  |
|  | - Altered sense of body | n=18 | - “I feel my own organ has been damaged and now a new one is available in my body. This new organ gives me a new life.” – Sheikhalipour (2018) |
|  | - Appreciation of life | n=17 | - “I felt like I received life again even once more . . . I have so much to live for, and now I feel I’ve moved back to life.” – Naden (2023) |
|  | - Body image | n=13 | - “I started putting on weight and I got an awful lot of hair on my face. I wanted to go out and look pretty but I couldn’t look pretty.” – Schipper (2014) |
|  | - Consideration of the donor (family) | n=21 | - “I thought it was a big decision for the person that’s donating their organs…I know they are getting something out of it as well; the person’s not died in vain, and I feel good about it.” – Kaba (2005) |
|  | - Distress | n=10 | - “I had great difﬁculties structuring and organizing my daily life. I quickly felt distressed and overwhelmed doing things that used to be easy for me.” – Seiler (2016) |
|  | - Emotional preparedness | n=12 | - “In my mind, I would undergo a transplantation that would settle everything, in my mind, the organ would be transplanted and life would go on and I wouldn’t have to worry.” – Bardet (2014) |
|  | - Facing death/trauma | n=15 | - “It’s [the transplant] a wonderful thing… It changes one’s life. It’s something like resurrecting a dead person.” – Tasdemir (2023) |
|  | - Fear of cancer | n=3 | - “I’m a hundred times more likely to get this cancer … and I’m two hundred and ﬁfty times more likely to get that cancer. So, that is worrying.” - Ó Lonargáin (2017) |
|  | - Fear of dialysis | n=9 | - “I can never forget the painful experience of…getting the dialysis done thrice a week, I am horrified by the sheer thought of returning back to that stage and wish to die before that.” – Kamran (2016) |
|  | - Fear of disease recurrence | n=4 | - **No quote** – “Despite improvements in their physical conditions after liver transplantation, they had fear over disease recurrence.” – Taher (2021) |
|  | - Fear of graft rejection/ failure | n=25 | - “The fear is that there is no hope. If you think too much about rejection, you become irritated…It is there. I brush it aside. And then I sometimes face it.” – Nilsson (2008) |
|  | - Fear of infections | n=9 | - “In the first six months, a year, I wore a mask almost all the time, and everyone who was visiting me had to have one. I was avoiding people, to go out; I was afraid.” – Petre (2021) |
|  | - Fear of medication side effects | n=5 | - “The long-term side effects like cancer worry me. I don’t like to go through a serious thing like this anymore. I don’t like to have this experience anymore.” – Pinter (2017) |
|  | - Fear of missed medication | n=3 | - “The medication was missing here and I am freaked out with the medication, because this one is against organ rejection too. Now I don’t know what’s going to happen.” – Santos (2016) |
|  | - Feeling vulnerable | n=12 | - “Why are they all fussing about me? You know, I am fine, I’ll be fine, maybe I am not fine?” – Wainwright (2018) |
|  | - Feelings of control/ helplessness | n=25 | - “If you have just a slight feeling that something is wrong you react very strongly because you are not familiar with what it is supposed to be like when you are transplanted.” – Almgren (2017) |
|  | - Feelings of isolation | n=11 | - “People in your surroundings think it’s all done now you’ve got a new kidney. And since then I’ve lost all the support from my surroundings.” – Schipper (2014) |
|  | - Feelings of peace | n=3 | - “I am no longer afraid of death. I have a peace about my mortality.” – Bean (2005) |
|  | - Future planning/changing priorities | n=13 | - “It is true that I live on borrowed time and I know that; It does not overshadow my everyday life, but of course I am aware that I am in no position to make plans for the next 10 years.” – Thomsen (2009) |
|  | - Future uncertainty | n=25 | - “It’s really tough…each day may be your last…I may suffer a rejection without knowing. I have to live with it for the rest of my life, I may die in a week, I may collapse here and now.” – Nilsson (2008) |
|  | - Gratitude | n=25 | - “I was very grateful; I’ll be indebted all my life to the chap that gave it to me and the surgeon that done it. Getting somebody else’s heart; it’s amazing.” – Kaba (2005) |
|  | - Guilt | n=21 | - “Maybe if I had not prayed and begged God to keep me alive, no one would have died so that I could survive.” – Bahramnezhad (2022) |
|  | - Health perceptions | n=22 | - “You’ll feel good sometimes, you’ll feel bad sometimes, it won’t all be bad, expect that you’re going to have a rollercoaster ride.” – Dudley (2007) |
|  | - Identity/worth | n=17 | - “Having a heart transplant does not mean that I am disabled, so it is important that I lead my life actively with my own identity.” – Yoo (2021) |
|  | - Low mood | n=13 | - “When I think I need to receive certain care services for life, I get fed up with life.” – Taher (2021) |
|  | - Mood swings | n=4 | - “Sometimes, I just have outbursts over trivial matters.” – Luk (2004) |
|  | - New possibilities | n=13 | - “It is different now, I understand that I can do any little thing at home. I help my wife, I work a little. I help out in a football school…I do various things!” – de Brito (2015) |
|  | - Optimism/hope | n=15 | - “I saw people who were in the same situation as me and I realised I wasn't alone and I saw that people who were approximately like me were OK, in other words, you could live.” – Petre (2021) |
|  | - Outlook on life | n=25 | - “I have come to the realization that if I want to live, I must take care of what I’ve got. I try with food and exercise to give my new lungs the best conditions.” – Graarup (2017) |
|  | - Patient burden | n=19 | - “Sometimes, you get tired, for example, every 2 months, you update medications, receive echocardiography and . . . It is a cycle.” – Peyrovi (2014) |
|  | - Perceptions of transplantation | n=8 | - “A miracle, an absolute miracle. A year has passed and my eyes are still filled with tears… May Allah attribute this to everyone who needs a transplant.” – Tasdemir (2023) |
|  | - Personality/mood changes | n=8 | - “My mood state is impaired. I have started fights with my wife all the time; I am angry at everything now, everything you can imagine.” – Fatma (2021) |
|  | - Religious/spiritual beliefs | n=8 | - “Early on, after my transplant, my faith in God increased, I became more religious…I always said thank you God! This heart is your divine gift to me.” – Pashaeypoor (2021) |
|  | - Second chance | n=20 | - “It’s pressure you put on yourself…to think, ‘Right I have to perform now. I have to live my life. I have to get on with things. I have to get a job…do this…get a family, kids.” – Ó Lonargáin (2017) |
|  | - Self-confidence/insecurities | n=9 | - “It feels as if it is I who have been neglectful rather than the body. You may feel that you have done something wrong even if you haven’t, it felt like that sometimes.” – Nilsson (2008) |
| ***Social functioning*** | | **n=37** |  |
|  | - Finding a new partner | n=6 | - “I have changed in such a way that I am no longer me…in sexual relations…the duration is half of what it was before…do you think it’s possible that I will ﬁnd a partner who accepts me like this?” – Abarca-Durán (2021) |
|  | - Interactions with other transplant recipients | n=14 | - “The wife couldn’t understand why I was like it and that, but obviously the stranger [other transplant recipient] could because she knew the experience of it all.” – Jones (2020) |
|  | - (Lack of) understanding from others | n=18 | - “Sometimes they say ‘get well soon’. ‘Get well soon’ is a nice thing to say, but they say it as if I have a very serious illness…I am healthy now.” – Tasdemir (2023) |
|  | - Medication interference | n=10 | - “When I’m invited out to dinner at someone’s house and that after a certain time I know that I have to stop because in two hours’ time I will need to take my medication.” – Bardet (2014) |
|  | - Relationship with partner | n=17 | - “My husband expects me to be the same as before but I am a bit anxious due to my physical changes…I wonder how can I have a normal married life?” – Kamran (2016) |
|  | - Relationships with employer/colleagues | n=5 | - “I talked to my manager…she said: “Oh well, you know, it’s hard for us to realise that you’re well now”… “We’ve probably been treating you a little bit different because you’ve been sick.” – Wainwright (2018) |
|  | - Relationships with family | n=24 | - “In the haemodialysis, you don’t have to withdraw from your family... But with the transplant, you have more time to dedicate yourself to it.” – de Brito (2015) |
|  | - Relationships with friends | n=10 | - “I don’t really take things for granted anymore, it makes you realise who your friends are, what sort of people you can rely on.” – Sargent (2007) |
|  | - Risk of infection | n=13 | - “It was like a quarantine for three months, very susceptible to infection…I had to travel with patient transportation, couldn’t take the tram or bus or hang out with anyone.” – Forsberg (2016) |
|  | - Risk of skin cancer | n=7 | - “I can’t really sunbathe anymore. I need to put at least a factor 25+ on. I’m quite young so its these things that I ﬁnd affect me the most.” – Sargent (2007) |
|  | - Social opportunities/ restrictions | n=19 | - “I can’t go to the beach. I can go but I have to stay in the shade, at most take a quick dip in the ocean and immediately go back to the shade. So why should I go anyway? I just don’t go.” – Santos (2016) |
| ***Role functioning*** | | **n=35** |  |
|  | - Ability to work | n=16 | - “But now I don’t know what will happen in October if it is decided that I am able to work 50% every day, that won’t be possible.” – Almgren (2017) |
|  | - Autonomy | n=15 | - “For me this was the greatest joy, to be free to drink water again, to be able to pee anytime, to eat fruits again. Oh my God! Such a joy!” – Petre (2021) |
|  | - Driving | n=2 | - “You can ride in a car, but you have to be careful. Let’s just say you can’t be in a car accident…you can have an accident and get hit right where the kidney is.” – Santos (2016) |
|  | - Financial implications | n=13 | - “They have said that they won’t allow me to go back to work. Then, what should we do? What about income? What will happen to our life?” – Dehghan-Nayeri (2017) |
|  | - Finding employment/ employability | n=8 | - “I applied for a job in a company and they asked me if I had any disease. I said I had a heart transplant and they immediately rejected me.” – Bahramnezhad (2022) |
|  | - Hobbies/leisure activities | n=11 | - “I go to all these places, buy all me screws and nails…and I do all these jobs…I couldn’t do any of that on dialysis.” – Ó Lonargáin (2017) |
|  | - Household tasks | n=4 | - “I cut the front lawn, the back lawn, did the hoovering and painted the garage door all in one day…I’m really pleased that I can do that sort of thing, which, before the transplant I was really limited.” – Boaz (2014) |
|  | - (In)dependence | n=14 | - “Today, I thoroughly enjoy that I can be there for my family. But I also enjoy that I can be independent again and I feel more like going out.” – Seiler (2016) |
|  | - Returning to ‘normal’ | n=20 | - “Feeling better is sometimes scary. You suddenly lose your daily routine. It was hard to ﬁnd new fulﬁlment in your days.” – Schipper (2014) |
|  | - Returning to work/changes in employment | n=16 | - “I resumed work full-time. But I was able to hold no more than 6 months.” – Bardet (2014) |
|  | - Self-care | n=4 | - “A bad day is when I hardly get dressed; a bad day is when I am in bed for most of the day, or lying on the couch … yes, I do eat, but that is because I have to.” – Thomsen (2009) |
|  | - Travelling | n=12 | - “Going on holiday can be problematic both due to potential illness and very high insurance rates: It’s just a bit of a bummer.” – Orr (2007) |
| ***Sexual functioning*** | | **n=12** |  |
|  | - Attitudes towards sexual activity | n=2 | - “When you…have received a kidney transplant, sex is not everything in a couple, there must be love and above all understanding.” – Abarca-Durán (2021) |
|  | - Interest in sexual activity | n=4 | - “I haven't felt the slightest bit interest in sex. Hugging but no more. It has been gone since the operation but now it’s back at last. It has been a great problem.” – Forsberg (2000) |
|  | - Reproduction/fertility | n=7 | - “The doctor told me several times not to have a plan for childbearing, because of taking many drugs.” – Peyrovi (2014) |
|  | - Sexual capability | n=4 | - “My sexual appetite has returned again and the hardness of my penis in sexual relations, if I complete the sexual act, it’s thanks to the transplant.” – Abarca-Durán (2021) |
|  | - Sexual fears/concerns | n=4 | - “You felt like a wound, an open wound only healed on the surface. Then you are not ready for intimacy. You want to protect yourself.” – Forsberg (2000) |
| ***Symptom distress*** | | **n=26** |  |
|  | - Breathlessness | n=2 | - **No quote** – “life with chronic rejection implied that reduced breathing might prevent the participants from other physical activities.” – Thomsen (2009) |
|  | - Changes in appetite | n=6 | - “After the transplantation…it took a while for hunger pangs to return to normal. But after that I was really ravenously hungry” – Beckmann (2022) |
|  | - Comorbidities | n=3 | - “Since all this colostomy business… I’ve aged considerably, but nothing to do with the kidney, more to do with the colostomy, the bowel thing.” – Pinter (2017) |
|  | - Gastrointestinal symptoms | n=7 | - “I had to change my anti-rejection drugs because of terrible diarrhoea.” – Sargent (2007) |
|  | - Hair growth/loss | n=12 | - “I have lost my long beautiful hair that I once used to have, and I am not slim anymore that makes me concerned and a bit upset too at times.” – Kamran (2016) |
|  | - Hypertension | n=3 | - **No quote –** “Several participants mentioned problems with…high blood pressure.” – Orr (2007) |
|  | - Infections | n=4 | - “It was a strain of pneumonia…supposed to be one of the worst ones for kidney transplants. Which I were worried cos me kidneys started ﬂuctuating a bit.” – Ó Lonargáin (2017) |
|  | - Musculoskeletal symptoms | n=2 | - “Unfortunately, this cortisone finished my bones and teeth” – Fatma (2021) |
|  | - Neurological symptoms | n=4 | - “My hands are shaking due to the drugs” – Fatma (2021) |
|  | - New onset diabetes | n=5 | - “I went through a stressful situation with the medication: I became diabetic.” – de Brito (2015) |
|  | - Oral symptoms | n=4 | - “I’m having a lot of problems swallowing, am burping a lot, and I can’t get the food down.” – Pinter (2017) |
|  | - Osteoporosis | n=4 | - **No quote –** “A range of conditions linked with their transplant including osteoporosis.” – Boaz (2014) |
|  | - Pain/discomfort | n=14 | - “I also had pain in my legs because of the medication…I couldn’t walk.” – Santos (2016) |
|  | - Scars | n=4 | - “I can still feel the scar, especially when sitting in the car or in my chair.” – Kristensen (2021) |
|  | - Skin symptoms | n=6 | - “I never thought about the side eﬀects until I started coming out in spots.” – Boaz (2014) |
|  | - Swelling | n=3 | - “After surgery, my face was getting bigger, and I was getting ugly and had hair loss, body swelling, scar on my chest.” – Yoo (2021) |
|  | - Urogenital symptoms | n=1 | - **No quote –** “Another developed ‘a lump inside my uterus’.” – Luk (2004) |
|  | - Visual impairment | n=1 | - **No quote –** “Symptoms included…deterioration in memory and vision.” – Luk (2004) |
|  | - Weight gain/loss | n=18 | - “Ate too much? Lounged around too much? No idea, I don’t know…All of a sudden it [my weight] was more.” – Beckmann (2022) |
| ***Fatigue*** | | **n=24** |  |
|  | - Chronic fatigue | n=10 | - **No quote** – “Patients still experience fatigue 6 months after kidney transplantation.” – Kristensen (2021) |
|  | - Energy/stamina | n=15 | - “I haven’t been working full time the last ten years. My energy is lost!...If I do a thing today, I wouldn’t manage anything tomorrow.” – Naden (2023) |
|  | - Tiredness | n=19 | - “I am very tired and need a lot of rest. After a busy day, I am so tired that I can hardly do anything.” – Seiler (2016) |
| ***Sleep*** | | **n=10** |  |
|  | - Hallucinations/dreams | n=3 | - “I had dreams of worms trying to eat me. I probably had these nightmares because of the medication.” – Eichenlaub (2021) |
|  | - Sleep difficulties | n=3 | - “All the medication dopes you – you are not yourself. And you feel really ‘crazy’ from not sleeping.” – Graarup (2017) |
|  | - Sleep disturbances | n=5 | - “I experience attacks of nighttime muscular spasm. Sometimes, the pain is so severe that it awakens me.” – Dehghan-Nayeri (2017) |
| *HRQoL = Health-related quality of life*  See *Supplementary table 5* for definitions of all health-related quality of life dimensions and outcomes and *Supplementary file_Outcome map* for the breakdown of outcomes identified by organ type.  Where explicit participant quotations were not available for an outcome, an excerpt from the results text of an included paper was provided as the supporting example. | | | |
